# Biomedical Potential of the Neglected Molluscivorous and Vermivorous *Conus* Species

**DOI:** 10.3390/md20020105

**Published:** 2022-01-27

**Authors:** Yihe Zhao, Agostinho Antunes

**Affiliations:** 1 CIIMAR/CIMAR, Interdisciplinary Centre of Marine and Environmental Research, University of Porto, Terminal de Cruzeiros do Porto de Leixões, Av. General Norton de Matos, s/n, 4450-208 Porto, Portugal; yihe.zhao@ciimar.up.pt; 2 Department of Biology, Faculty of Sciences, University of Porto, Rua do Campo Alegre, s/n, 4169-007 Porto, Portugal

**Keywords:** *Conus*, feeding habit, phylogenetic tree, distribution map, biomedical compounds

## Abstract

Within the Conidae family, the piscivorous *Conus* species have been a hotspot target for drug discovery. Here, we assess the relevance of *Conus* and their other feeding habits, and thus under distinctive evolutionary constraints, to highlight the potential of neglected molluscivorous and vermivorous species in biomedical research and pharmaceutical industry. By singling out the areas with inadequate *Conus* disquisition, such as the Tamil Nadu Coast and the Andaman Islands, research resources can be expanded and better protected through awareness. In this study, 728 *Conus* species and 190 species from three other genera (1 from *Californiconus*, 159 from *Conasprella* and 30 from *Profundiconus*) in the Conidae family are assessed. The phylogenetic relationships of the Conidae species are determined and their known feeding habits superimposed. The worm-hunting species appeared first, and later the mollusc- and fish-hunting species were derived independently in the Neogene period (around 23 million years ago). Interestingly, many *Conus* species in the warm and shallow waters become polyphagous, allowing them to hunt both fish and worms, given the opportunities. Such newly gained trait is multi originated. This is controversial, given the traditional idea that most *Conus* species are specialized to hunt certain prey categories. However, it shows the functional complexity and great potential of conopeptides from some worm-eating species. Pharmaceutical attempts and relevant omics data have been differentially obtained. Indeed, data from the fish-hunting species receive strong preference over the worm-hunting ones. Expectedly, conopeptides from the fish-hunting species are believed to include the most potential candidates for biomedical research. Our work revisits major findings throughout the *Conus* evolution and emphasizes the importance of increasing omics surveys complemented with further behavior observation studies. Hence, we claim that *Conus* species and their feeding habits are equally important, highlighting many places left for *Conus* exploration worldwide. We also discuss the Conotoxin drug discovery potentials and the urgency of protecting the bioresources of *Conus* species. In particular, some vermivorous species have demonstrated great potential in malaria therapy, while other conotoxins from several worm- and mollusc-eating species exhibited explicit correlation with SARS-CoV-2. Reclaiming idle data with new perspectives could also promote interdisciplinary studies in both virological and toxicological fields.

## 1. Introduction

It is generally believed that more than 700 *Conus* species have evolved during the last 50 million years [1,2]. With the highest species abundance occurring in southeast Asia, most *Conus* can be found in the shallow waters of tropical and subtropical oceans [3]. *Conus* is widely distributed in rocky shores, sandy beaches, coral reefs and intertidal waters, with depths reaching up to over 600 m [4]. However, geophysical environment is not the only factor that influences the *Conus* species distribution. For example, despite similar oceanic and climate conditions, more than 77 *Conus* species have been discovered in Indian coastal waters, especially in the Tamil Nadu Coast and the Andaman Islands [4,5,6], with fewer than 20 species reported on the eastern side of the Andaman Sea [7]. Nowadays, *Conus* species are generally overexploited, and some species are now endangered. Exploring these waters of potentially high species diversity could enrich our understanding of their population’s genetic structure and provide the missing pieces for clarifying *Conus* evolution. As the conotoxin compounds vary greatly throughout the growth stages and across geolocations within the same species, further investigation of these species-specific regional distribution differences may provide crucial insights for artificial breeding and harvesting specific bioactive compounds in the future.

With intraspecies variation of the venom cocktails, a huge array of remarkably diverse conopeptides, ranging from 50,000 to 140,000 venom peptides, are estimated to be produced in the *Conus* genus [8]. With a unique repertoire of 100–200 venom peptides for each species, *Conus* could presumably specifically target a wide range of vertebrate and invertebrate physiological receptors, assisting their prey hunting or defense from potential predators and competitors [9]. Different prey targets come along with different venom recipes. Yet, despite the species diversity of *Conus*, their feeding habits can be categorized into three main types, often with associated differences in the radula morphology [6,10]. The radula of the vermivorous (V) *Conus* usually have two barbs or the second barb replaced by a blade; spur and serration are present, with or without waist. Spur is absent in the molluscivous (M) radula; serrations are longer and terminate at a prominent cusp. The radula of the piscivorous (P) *Conus* has three barbs, with a recurved tip on the third barb with no serration, waist, cusp or spur [6]. Exceptionally, in some vermivore species, the radula was found to be similar to the molluscivous radula, indicating a more complex relationship between the radula morphology and feeding habit [11].

Alongside radula changes, *Conus* also developed complex venom compositions and envenom system. The venom gland of the sea snails can be divided into three sections: bulb, duct and radular sac [12]. To hunt and defend themselves, each *Conus* species can produce a cocktail of conotoxins (reaching hundreds of small polypeptides with very complex post-transcriptional modifications, alongside some paralytic small molecules) in their venom glands and inject the precise amount of venom cocktail through the duct anchored on its prey by the harpoon [13,14,15]. The prey will be instantly paralyzed through rapid stabbing with such an efficient venom cocktail [16]. Previous studies also proposed that modern *Conus* species can produce venom peptides with different functions at different parts of the venom gland, even for different parts of the venom duct. For example, the distinct defense cabal was identified from bulb-proximal and predation-evoked cabal from the bulb-distal end of the specialized venom ducts [17,18]. The diverse conotoxins allow *Conus* to precisely adjust their cocktail recipes for each attack (hunting, defending or threatening competitors) [19] or become specialized in a local prey according to geographic variation [20]. Recent studies further illustrated that the defensive compositions of venom cocktails of the worm-hunting species may be the driving factor for the emergences of fish- and mollusc-hunting species [21,22]. Consequently, conotoxin compositions are also expected to vary from lab culture to natural conditions.

In general, the small polypeptides (mostly 10 to 40 amino acid residues) are rich in disulphide bonds and act on the nervous system of the target organism, interfering with signal transmission in cells and neurons [23]. Specific types of cysteine residues and disulphide bond arrangements have been found in different gene superfamilies, with each venom gene superfamily usually having unique corresponding binding sites from different pharmacological target families (Table 1) [3,24]. The targets of conotoxins include: (i) presynaptic membrane calcium channel or G protein receptor, (ii) voltage-gated potassium channel, (iii) adrenal hormone receptor, (iv) serotonin receptor, (v) somatostatin receptor, (vi) norepinephrine receptor, (vii) voltage-gated calcium ion channels and (viii) other targets [25,26]. These receptors are ubiquitous when it comes to drug research. Conopeptides, with their abundant diversity, novel chemical structure, biological sensitivity and target selectiveness, are of great potential as drug precursors, as well as an important neuroscience tool [27]. 

Additionally, nerve system agents and anticancer drugs are research hotspots, and conotoxins are drawing more attention after the entry of Ziconotide (Prialt^®^) on the market [30]. Because the physiological system of fish is more similar to other vertebrates, such as human [31], in comparison to benthic organisms, such as worms and mollusks, many studies focus exclusively on the piscivorous species and their venom production. To better categorize the complex conopeptides, the primary structure of 222 conotoxins has been identified and classified into several gene superfamilies, according to precursor signal peptides and amino acid sequences [25,32]. Apart from their genetic diversity, the precursor RNA splicing process contributes greatly to the conotoxin diversity. With just over 30 gene superfamilies, more than 7000 conopeptides have been discovered [33,34,35]. Not all of these peptides, however, are of equal importance. Compounds from A, M, O1 and T gene superfamilies account for the bulk of venom cocktails. Moreover, the expression of some conopeptides is so low that their presence cannot even be detected in traditional proteomic experiments [18,36]. Perhaps it is the seemingly redundant venom compounds that are providing abundant raw materials for natural selection and contributing to the rapid speciation and quick adaptation of the *Conus*. Therefore, to understand the rich proteomic and toxicological resources in *Conus*, genomic and transcriptomic approaches are required to assist conotoxin identification and classification [37], as they are more sensitive and accurate for capturing the trace amount of conopeptides. Hence, the biggest obstacles now hindering the use of multi-omic approaches in *Conus* studies are data analysis and the scarcity of well-annotated genomes.

Although increasingly more *Conus* transcriptomes have been sequenced, the quality of these datasets is hardly consistent. Interpretations of the assembled *Conus* genomes are still at an early stage. Before 2020, only fragmented genome data of three *Conus* species were available: a highly fragmented assembly from *Conus bullatus* (P/M) with total assembled length of only 201 Mb [38]; *Conus tribblei* (V) with total assembled length of 2160.5 Mb and N50 of 2681 bp; and *Conus consors* (P) assembly of 2049 Mb with an N50 size of 1128 bp, regardless of the estimated genome size for *Conus consors*, which was 3.025 Gb, based on K-mer distribution [39]. Apart from the species mentioned, an estimated genome size for *Conus tribblei* (V) was 2.76 Gb [40], 2.56 Gb for *Conus bullatus* [38], 3.60 Gb for *Conus pennaceus* (M) and 3.90 Gb for *Conus lividus* (V) [41]. Recently, several publications have released well-assembled *Conus* genomes based on more advanced sequencing technologies. The first intact genome assembled was retrieved from *Conus betulinus* (V). The assembly was 3.43  Gb in size, which is slightly smaller than the estimated genome size of 3.99 Gb (86%) from previous fluorometric assays. Overall, 35 large groups of superscaffolds have been constructed from the clean Hi-C reads [42]. *Conus ventricosus* (V) follows closely by, with a 3.59 Gb chromosome-level genome, which represents 87.6% of a flow cytometry estimated haploid genome size (4.1 Gb). Similarly, 35 largest scaffolds or pseudochromosomes were identified from *Conus ventricosus* [43]. It is not negligible to notice that these two new genome assemblies are both smaller than their genome size estimation and yet bigger than the genome size estimation of other *Conus* species. Gastropods usually also have a wide range of chromosome numbers [44]. Regarding the Conidae chromosomes, they vary from 16 pairs in *Conus magus* (P) [45] to 35 pairs in *Conus coronatus* (V) [46]. Despite the high heterozygosity of *Conus* genomes, a few *Conus ventricosus* (V) specimens showed 34, 35 or 37 chromosomes, although their haploid number was estimated to be 36 [47]. Therefore, for further research, it would be important to collect various omics data from the same specimens. As *Conus* is a fast-evolving and highly diversified genus, specimens from different populations may result in very different omics outcomes. Apart from genetic variations between populations, metabolism and transcription factors regulating their venom expression can be poles apart. With a good transcriptome and genome dataset, the evolutionary path can be further illustrated. The “fossils” of conopeptides genes can be studied from pseudogenes. Most details of regulatory factors, introns and post transcriptome modifications of each venom gene superfamilies can be examined like never before. The mechanism behind conotoxin evolution and the species’ evolutionary history could now be discovered by the popularization of tools in multi-omics and increasingly more integrated multi-omic data.

Here, we sum up some main outputs in the field of *Conus* studies from past years, providing a clearer outlook in the scope of *Conus* feeding habits, distribution, evolution and conservation status. We emphasize the importance of reviewing the neglected *Conus* datasets in biomedical investigations and optimize the use of these prominent sources. Based on this structured knowledge, this work connects the COVID-19 studies to the venoms, and it will enable interdisciplinary researchers to quickly learn more about the field of *Conus*.

## 2. Results and Discussion

### 2.1. Feeding Habits and Evolution Path

Apart from the 190 species from three other genera (1 from *Californiconus*, 159 from *Conasprella* and 30 from *Profundiconus*) in the Conidae family, there are currently 728 species recognized in the *Conus* genus, making it one of the most diversified species in the Conoidea superfamily (Supplementary Table S1). In this study, the feeding habits of known species are categorized based on radula morphology, field reports and dissection of digestive chambers. Only 506 species in the Conidae family were classified into the staple feeding habits. For *Conus*, it is obvious that worm eating is the dominant feeding behavior, accounting for more than 72% of total species diversity, including most basal species, and thus likely to have first emerged in their evolutionary history. This trend was followed by the most recent fish-eating group (20%) and molluscivore group (12%). Interestingly, several polyphagous species have also been found scattered among the three monophagous groups, indicating a more complex hunting behavior of the *Conus* species in natural environments. 

**Figure 1:**
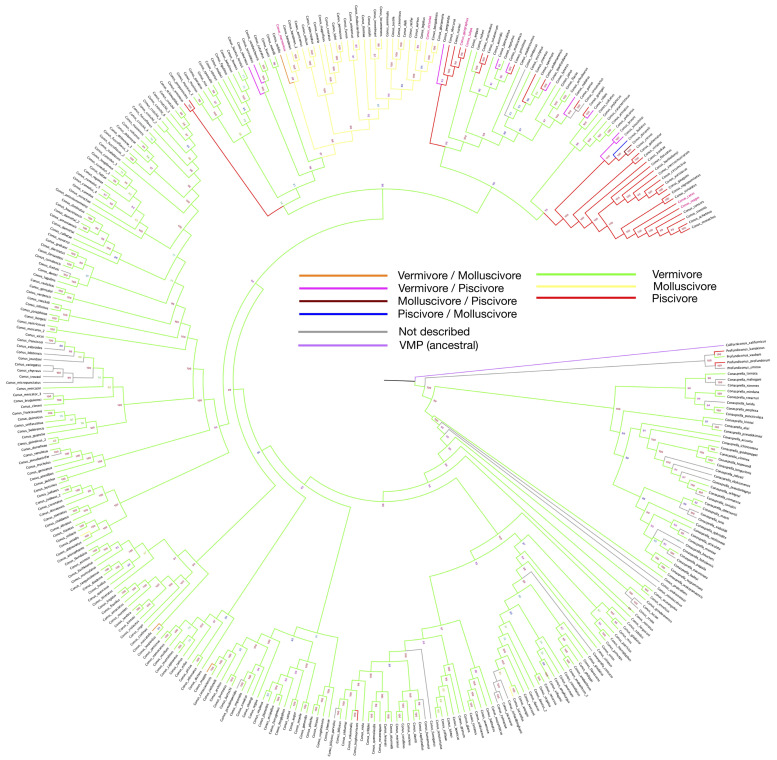
Phylogeny tree of 335 *Conus* species, constructed by using barcode sequences (12S, 16S and COI). Branch colors are set according to the feeding habit from Supplementary Table S1. Bootstrap values are presented by the numbers next to each branch. Species with feeding habits found have been assigned with branch colors accordingly. Names of species that have been involved in drug development are labeled in red.

The phylogeny tree constructed with barcode sequences (12S, 16S and COI) is consistent with previous studies, and we further highlighted the feeding habits using a color-coded pattern across the various species studied here with the same feeding habits (Figure 1). As shown in the phylogenetic tree, following the vermivorous group, the first batch of fish-hunting species appeared in the Neogene period (around 23 million years ago) [48]. Unlike the cluster of molluscivorous *Conus* species that share one collective root on the phylogenetic tree, the emergence of the piscivores seems to be independent and recurrent along with speciation.

According to the latest taxonomy update from the WoRMS website, several species have been categorized into the same species, as indicated by trailers in the graph (Figure 1). As shown in the tree, polyphagy is most likely to occur on restricted branches, where fish-hunting species have secondarily recurrently emerged from the worm-eating species. As a true omnivore, *Californiconus californicus* not only feeds on marine worms, fish and mollusks; it is also a scavenger. Positioned on the proximal branch of the phylogenetic root, the mentioned *Californiconus californicus* is considered as an ancestral state species to others in the family. Therefore, it is interesting to see that the majority of *Conus* species become specialized in only one particular prey category, which makes the few polyphagous species outliers, or more interestingly, transitive species.

Many studies have suggested that the venom cocktail composition for each preference is highly distinguishable. The venoms of fish-hunting species are well selected to target vertebrate nervous system, in which receptors are very different from invertebrates. If we look at *Conus* alone, vermivores are obviously plesiomorphic, and molluscivores and piscivores originate from them. Furthermore, the distinguishable evolutionary patterns between the fish- and mollusc-hunting species indicate that the determining factors for different feeding habits may be separately located in the genomes, where gene silencing or gene lost mechanism could play an important role. Otherwise, it would be difficult to explain the seemingly random occurrences of the fish-hunting species. 

However, there are also cases of one *Conus* species hunting various preys, or even some individual showing omnivorous behavior. This suggests that *Conus* feeding habit is not monophagous and far more complex than simple hunting/defense venom recipes. Indeed, other factors, such as environmental pressure and prey abundance, could also play important roles in venom secretion. 

*Conus* evolution is subject to both convergence and divergence. Different groups in *Conus* could share similar evolutionary patterns due to comparable selection pressure in a confined geographical region. Previous molecular phylogenetic analysis has confirmed that *Conus* species constitute a largely heterogeneous group, despite their overall morphological homogeneity [49]. This may explain that, although the selective pressure on the venom is acting in different directions, vermivorous species, which are close to the fish-hunting clade, show great potential in accepting both worm and fish as prey. 

On the phylogeny tree, some vermivorous sister species exhibit a fish-eating behavior or can also feed on other molluscs, suggesting a loose hunting strategy. Given that the roots of most divergent species are very recent, it indicates an ongoing adaptive process of some *Conus* clusters [50]. This phenomenon could be caused by both genetic plasticity and environmental pressure. It is also intriguing that fish-hunting and mollusc-hunting strategies were both developed at the same period of geological time, and the two clusters are genetically close to each other.

The origin of the Conidae family dates back to around 59 (73–55) million years ago, and the radiation of some analyzed species within the *Conus* genus was estimated to have occurred between 24–15 (30–12) million years ago [51]. The explosive fish speciation movement [52] in this period (Neogene) became the ultimate driving force and selection pressure for some clades of *Conus*. The emerge of massive Pleistomollusca species [53] occupied ecological niches and stiffened the competition, potentially leading to the clade of molluscivores.

However, questions remain on the *Conus* feeding habits. Normally, most specimens were dissected right after being collected for further multi-omics studies, and stomach contents and radula shapes were kept for feeding habit analysis. Only a small number of individual species have been kept for feeding behavior studies. The phylogeny and feeding habit results described in this work suggest a more complicated relationship between feeding habits and morphology.

Replicates are rare for most transcriptome datasets online, and gene expression can be influenced by environmental conditions, individual sample difference and other batch effects, making it challenging to compare datasets from various projects. Therefore, designed experiments should be carried out to retrieve precise transcriptome data for different tissues, and most importantly, the venom glands. Further studies could also explore venom cocktail composition difference induced by different pressures, such as constant defensive pressure, and how *Conus* would cope with the pressure when food options are limited, especially for the polyphagous clades labeled on the phylogenetic tree (Figure 1).

### 2.2. Distribution

Geographical coordinates of the *Conus* species from the last clade on the phylogeny tree are pinpointed on the map. On the map, *Conus* species that prey on both worm and fish (V+P) are spread largely in the shallow waters of southeast Asia, Australia and African east coast, corresponding to the distribution areas of the most diversified *Conus* species [3]. Apart from ocean currents, which may prevent the species from dispersing globally, the distribution pattern of V+P species could result from a high competitive pressure and diversity of potential food sources in these areas. The geographical landform of islands has also accelerated evolutionary diversification [50]. 

**Figure 2:**
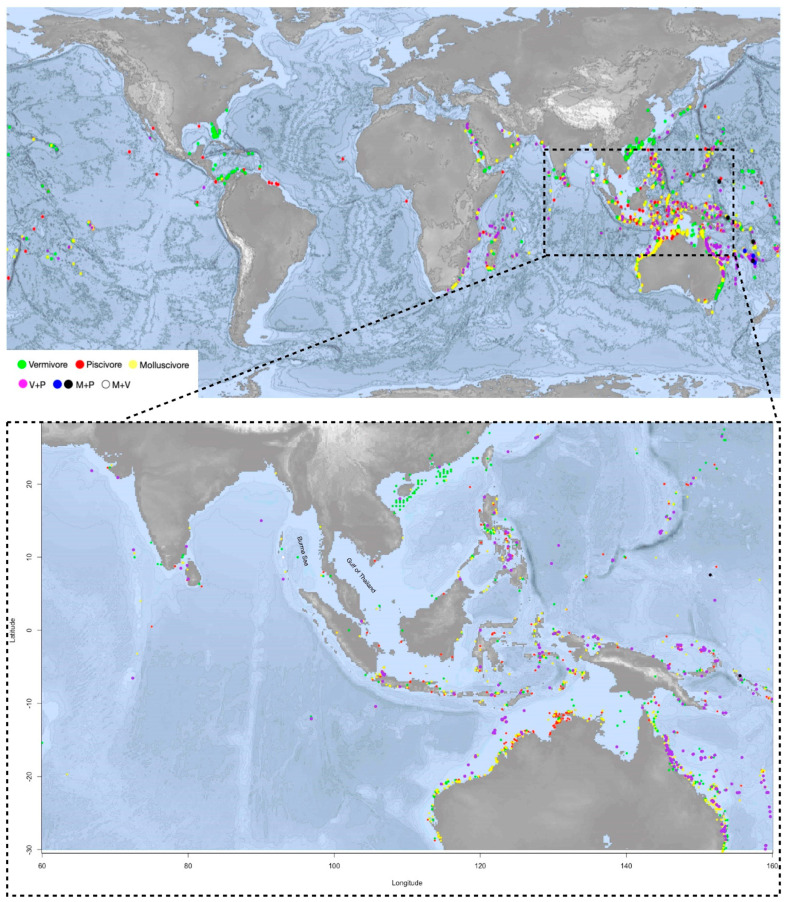
The upper map shows the distribution of the monophagous and polyphagous *Conus* species clades from the phylogenetic tree (species listed in the Supplementary Table S2). Orange for molluscivous (M), red for piscivorous (P), green for vermivorous (V). Purple-color dots indicate the feeding habit of V+P (vermivorous and piscivorous); blue and black dots are for M+P (molluscivous and piscivorous); and the white dot is V+M (vermivorous and molluscivous). *Conus* species V+P are spread largely in the shallow waters of southeast Asia and Australia and African east coast, corresponding to the distribution areas of the most diversified *Conus* species. The distribution pattern of V+P species could result from a high competitive pressure and diversity of potential food sources in these areas. The lower map highlights the region where *Conus* species are most abundant and diversified, including the north coast of Australia and south and southeast Asia.

Interestingly, despite geological and hydrologic conditions being very similar to the surrounding waters, in south and southeast Asia, comprising the most species-rich area, few *Conus* species have been reported on the east coast of the Burma (or Andaman) Sea and the Gulf of Thailand (Figure 2). Little research and few scientific reports have been conducted in these areas, which could flag a potentially species-rich region for further studies.

### 2.3. Conservation Status

Despite *Conus* resources being relatively abundant, it is of great ecological and biological necessity to protect them. Shell hunting of the *Conus* has already driven some of the rare species to the edge of extinction. According to the International Union for the Conservation of Nature (IUCN) Red List, 26 *Conus* species are Near Threatened, 27 species are Vulnerable, and 14 more species have already been Endangered or Critically Endangered, accounting for 9.2% of the total *Conus* species globally. The number seems to be relatively small, but some regions are suffering huge depletion. For instance, all three of the Critically Endangered species, along with another four (out of 11) Endangered species, are located in the Cape Verde waters; 45.3% of the 53 species are assessed as Threatened or Near Threatened with extinction. It is shocking to find that 61% of the 41 *Conus* species are threatened with extinction in the west Africa area [54,55]. Shell hunting and habitat degradation are the main threatening factors for the *Conus*. Human activities, such as tourism and industrial and residential effluent pollution, have severely affected local *Conus* communities. In west Africa, there has been an observable reduction in species abundance and body size.

### 2.4. Drug Discovery

Venomous animals are widely distributed in the biosphere, and over time, a positive selection between venom and its target has been driven by the predator and prey arms race. Venoms are usually composed of novel chemical structures with various targets. Therefore, they encompass a pool of broad molecular structures highly relevant for novel drug discovery.

Marine toxins are one of the most rapidly growing research areas in bioactive substances. As unique chemical structures are more often found in venoms, the structure–function relationship of marine toxins is a valuable clue for discovering new targets and pathways. The most common marine toxins can be found in pufferfish and crabs; some toxic shellfish can also produce paralytic neurotoxins. Polyether toxins can be easily transmitted to humans through the food chain and cause food poisoning, such as palytoxin, dinophysistoxin and ciguatoxin. Other marine toxins include alkaloids, for example, saxitoxin. Among all the marine toxins, polypeptide toxins are so far the most effective and deadly (tetrodotoxin and conotoxin). They are usually highly selective with the nervous system, digestive system, cardiovascular system and cell membrane, with especially strong cardiotoxicity and cytotoxicity. This makes venom peptides very prominent for drug discovery. As a widely studied marine invertebrate, its diversified conopeptides make *Conus* promising animals for the discovery of antimicrobial peptides, analgesics and anti-cancer drugs. 

**Figure 3:**
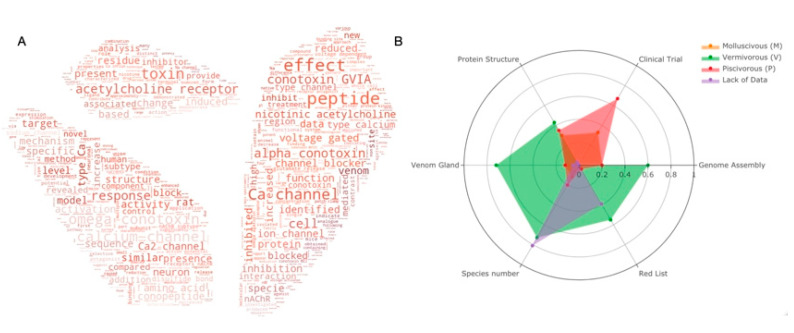
(**A**) Keyword cloud from the abstract of *Conus* studies over the past 20 years; (**B**) Statistics of current *Conus* studies. Ratio of six categories (genome assemblies, species under clinical trials, characterized protein structures, venom glands, species number and red list species) for *Conus* species of each feeding habit are presented in the radar plot. Orange for molluscivous (M), red for piscivorous (P), green for vermivorous (V) and species with insufficient data are presented in purple. Species with more than one feeding habit are counted separately in each group. Raw statistics can be seen in Supplementary Tables S3 and S4.

Many works have committed to decipher the secrets of conopeptides. Figure 3A shows the top 100 keywords in the field for the past two decades. It is obvious to see that most publications are related to the ion channels (Ca^2+^, sodium, voltage gated) and peptides–receptor interactions (acetylcholine receptor, inhibition, block). The most studied conotoxins are from alpha and omega pharmaceutical families. Protein structures and animal-trials-related topics are less reported.

As mentioned, worm-eating *Conus* species (V) is the largest group of the three genera. It also has the highest number of venom glands (31 out of 43 transcriptomes, Supplementary Table S4) and genome assemblies (2 out of 3), and it is the most endangered species. In contrast, there is no genome assembly so far in the mollusc-hunting group. Even though most of the data are for the worm-hunting species, mollusc- and fish-hunting species are more favored in clinical research. It is notable that a number of species are in danger with little data reported.

In the past 20 years, with Ziconotide being approved by the FDA, a number of conotoxins have been selected for clinical research (Table 2). This is justified, since the prey for piscivorous *Conus* species is vertebrate, whose physiology system is more similar to the human system. Therefore, most drug-related research has focused on this group. Interestingly, mollusc-hunting *Conus* sourced molecules are also seen on the list, even if their prey is invertebrate. An important question to investigate now is whether the worm-hunting species, believed to be less promising for drug discovery, underestimated.

The high relevance between venom peptides and the human nervous system is attention calling. Publications on other venomous animals have shown significant potential in antibacterials, cancer therapy, nervous system and cardiovascular diseases [56,57,58,59,60,61,62,63]. Given the vast application of other venoms, conopeptides are very under-explored and of such great potential. Yet, it remains difficult to isolate the most effective molecules with minimal side effects. Apart from Prialt, all other candidates present many unaddressed issues.

### 2.5. Structural and Functional Studies of Conotoxins with Their Receptors

The realization of conotoxins application in the pharmacological industry depends heavily on the structural and functional studies of conotoxins with their receptors. The physiological functions of conotoxins are fairly well understood, although the well-defined functions and structures account for only a small part of the entire toxin pool. The difference in cysteine skeletons and peptide structures determines the reactive sites of conotoxins. The identified targets mainly include ligand-gated ion channels (acetylcholine receptors, NMDA receptors), voltage-gated ion channels (sodium, potassium, calcium channels) and G protein-coupled receptors.

Acetylcholine receptors (AChRs) are widely expressed in the nervous systems, playing critical roles in various physiological processes. nAChRs are associated with various neurological diseases, such as Parkinson’s disease, Alzheimer’s disease, neuralgia and epilepsy [66]. According to pharmacological activities, AChRs are classified into nicotinic (nAChR) and muscarinic (mAChR) types. α-conotoxins have become an ideal tool for studying nAChR. They usually contain two loop structures formed by two pairs of conservative disulphide bonds (except for three pairs for α-SII) and can selectively inhibit neuronal or muscle-type nAChRs. There are three known conotoxin families (α-, αA- and ψ- conotoxins), which can selectively react to nAChRs. For example, conotoxin Vc1.1 has significant analgesic activity for nAChR rat subtype α9α10 [67]. It has been used as an analgesic and entered clinical phase II study [68,69]. However, the project was eventually terminated due to low affinity for the human subtype [70]. On the other hand, GeXIVA is in the preclinical stage as an analgesic for the neuralgia treatment, indicating that α9α10 nAChR blockers have a good prospect for the development of analgesia [71]. Other prominent compound target at nAChRs includes recently discovered octaoligoarginine R8, α-conotoxins RgIA and previously isolated LvIA and LvIF from *Conus lividus* (V) [66,72,73,74]. In particular, LvIA and LvIF are being intensively studied, have exhibited great binding ability to the α3β2 nAChR receptor and have great potential as new candidate tools for studying α3β2 nAChR-related neurophysiology and pharmacology [66,72].

Conotoxin is also an ideal tool for studying ion channels and membrane receptors. For example, ω-GVIA has become an identification tool for N-type voltage-gated calcium ion channels [26]. Conantokin, as a highly selective antagonist of NMDA receptors, is expected to be a promising candidate for the treatment of epilepsy.

In short, conotoxin drug discovery depends on the variation of conopeptides. Fortunately, there is a large number of *Conus* species and an even larger amount of conopeptides that remain to be filtered. For the vermivorous species, there are far more data than for the other two groups to investigate. Multi-omics data provide a great scope for discovering novel mechanisms and pathways, and there is a hidden value for worm-hunting species in drug discovery. On the other hand, a considerable number of *Conus* species are facing survival difficulties. If they become extinct, venom peptides diversity will decrease as well. So far, only few *Conus* species have been thoroughly studied, and some of them are closely related (Figure 1).

### 2.6. Potential Value in COVID-19 and Other Diseases

Recently, one of the vermivorous species, *Conus nux*, has shown breakthrough from a different research angle. A newly published study suggested an anti-adhesion adjunct therapy against malaria and the potential of conotoxins in mitigating, or even curing, fatal diseases, such as AIDS and COVID-19, as some of the conopeptides are potential inhibitors of the specific protein of these viruses [75]. 

Furthermore, an intriguing correlation has been found between the toxin-like peptides and the samples collected from COVID-19 patients. Among all the peptides identified, conotoxin-like peptides are ubiquitous: sequences from *Conus tulipa* (P), *Conus geographus* (P), *Conus marmoreus* (M), *Conus radiatus* (V/P), *Conus virgo* (V), *Conus quercinus* (V), *Conus pulicarius* (V), *Conus flavidus* (V), *Conus catus* (V) and *Californiconus californicus* (V/M/P) have been identified in plasma, urine and fecal samples of COVID-19 patients [76]. This might explain the occurrence of many neurotic related symptoms observed in some COVID-19 patients, such as hyposmia, hypogeusia and signs typical of the Guillain–Barre syndrome. Some of the conotoxins target ion channels and nicotinic acetylcholine receptors, causing dysfunction of the receptors, and alter acetylcholine levels. Furthermore, a potential interaction between spike protein of the SARS-CoV-2 and nicotinic acetylcholine receptors has been reported [77]. Understanding how the different nAChRs are affected by the S-peptide and acetylcholine-related conopeptides may be relevant to uncovering COVID-19 pathophysiology. Therefore, if we know that the produced conotoxin-like molecules can cause the symptoms, it also provides insight into finding clues for neutralizing antibodies in the *Conus*-related studies. The coevolution between the *Conus* and their prey, which has existed for a long time, may also warrant further research. By studying the prey, there might be a chance to discover a coping strategy and then use it on patients.

So far, the findings from the worm- and mollusc-eating *Conus* species have shown a direct correlation with current global health emergencies. In future studies, with more conotoxin 3D structures constructed and protein–protein interaction simulation improved, the potential for developing medical treatments and pharmaceutical molecular tools from the worm- and mollusc-eating species, alongside the fish-hunting ones, is enormous.

## 3. Materials and Methods

In this review paper, the procedures involved in producing the figures and tables are described in further detail below.

### 3.1. Species and Feeding Habits

*Conus* species names were collected from WoRMS (http://www.marinespecies.org, 6 June 2019) and sorted mainly according to the up-to-date speciation standard [2]. A total number of 931 species from the Conidae family were classified into four genera. Then, *Conus* species were further categorized into several different feeding habits, based on their morphological characters and observations documented in other studies.

### 3.2. Geographic Coordinates Information

Coordinates for *Conus* species were downloaded and filtered from three resources: Atlas of Living Australia (https://www.ala.org.au, accessed on 6 June 2019), BOLD system (http://www.boldsystems.org, accessed on 6 June 2019) and OBIS-Ocean Biographical Information System (https://obis.org, accessed on 6 June 2019). After combining all three databases, geographical distribution of *Conus* species on the world map was presented by “marmap” package in RStudio.

### 3.3. Sequence Alignment and Phylogenetic Analyses 

Barcode sequences (12S, 16S, COI) were downloaded from NCBI and integrated using “Genius”. All sequences mentioned can be found in Table S1. Nucleotide sequences were aligned using MEGA7 built-in program Clustal W [78]. Aligned regions where homology could not be guaranteed were removed manually. Model GTR + F + R9 and 1000 bootstraps were selected for Maximum Likelihood phylogenetic tree reconstruction by IQ-TREE [79]. FigTree v1.4.4 (http://tree.bio.ed.ac.uk/software/figtree/, accessed on 11 December 2021) was used for visualization and annotation of the constructed phylogeny tree.

## 4. Conclusions

Our findings shed light on *Conus* evolution and distribution. *Conus* genus is widely distributed globally, yet there are many places left for exploration, such as the west coast of Africa, the Indian Ocean, the littoral areas of the Mediterranean Sea and even southeast Asia. Conotoxins are highly diverse and evolve at a fast rate, and they have great potential for the development of new drugs. Due to high gene diversity and morphological confusability, many newly discovered species are to be confirmed [80]. Moreover, the *Conus* vacuum on the east coast of the Burma (or Andaman) Sea and the Gulf of Thailand shows that there is still enormous and untapped potential to be uncovered. Furthermore, behavior observation and feeding habit information for the most species remain unknown, and they are important to understand the *Conus* evolution.

In this study, we revisited the published barcode data of 335 species and combined them with feeding habits and geological information to deduce related phylogenetic inferences. Our study results have confirmed that both fish- and mollusc-hunting behaviors are derived from vermivorous ancestors. It is likely that molluscivous species originated from a single root, while the origin of the piscivores is polytopic. Interestingly, the phylogenetic tree and the distribution map show that a number of species in the warm and shallow waters have evolved to prey on both fish and worms. This newly gained trait is multi originated. This is controversial to the traditional idea that most *Conus* species are specialized to hunt certain prey categories. It shows the complexity and great potential of conopeptides from some worm-eating species.

Although we have more *Conus* data published every year, the quality of each transcriptome varies, and some transcriptomes are less than satisfactory. Now, with two more complete genome assemblies available, further *Conus* genome assemblies of some closely related species can be facilitated. Notably, complete genomes of mollusc- and fish-hunting *Conus* genome are still absent, limiting a lot of drug research to its primary stage. It has long been believed that only animals with features related to human features are of great or direct value. This led to our current contradiction that all *Conus* researchers are facing: the most abundant species and well-sequenced specimens are not the ones we want to study most. On the contrary, with newly published genome assemblies and more published omics data every year, it is an opportunity for proposing a deeper investigation of the neglected *Conus* species. Since vermivores are plesiomorphic, and the whole-genome duplication event has been confirmed [43], it is worth to reassess the values of worm- and mollusc-hunting *Conus* species in evolutionary and venom composition studies complemented with transcriptomic and proteomic assessments.

For future omics studies, it is important that data should be collected from the same individual species, so as to reduce uncertainty during analysis. From the evidence provided, it is obvious that all *Conus* species are equally important and directly related to our lives. Compared to the fish-hunting species, the worm-eating and mollusc-hunting *Conus* species retain a larger conotoxin reservoir. Correspondingly, recent studies on the anti-adhesion adjunct therapy against malaria and the close correlation with some current global health emergencies, such as AIDS and COVID-19, have proved the indispensable contribution of the once-neglected species. Reclaiming the idle data with new perspectives could also promote interdisciplinary studies in both virological and toxicological fields. 

In summary, the abundant transcriptome and proteomic data on the worm- and mollusc-hunting species provide great opportunities for evolutionary analysis and are of tremendous pharmaceutical potential. With increasingly more conotoxin 3D structures being constructed and simulation power accelerated, developing medical treatments and pharmaceutical molecular tools from conotoxins and their receptors could be envisioned.

## Figures and Tables

**Table 1:** Conopeptides gene superfamilies, cysteine framework and their pharmacological families [3,28,29].

Conotoxin Family	Definition	Gene Superfamily	Cysteine Framework
α (ALPHA)	Nicotinic acetylcholine receptor (nAChR)	A, B3, D, J, L, M, S	I, II, III, IV, VIII, XIV, XX, XXV
γ (GAMMA)	Neuronal pacemaker cation currents (inward cation current)	O1, O2	VI/VII
δ (DELTA)	Voltage-gated Na Channel (agonist, delay inactivation)	O1	VI/VII
ε (EPISILON)	Presynaptic calcium channels or G protein-coupled presynaptic receptor	T	V
I (IOTA)	Voltage-gated Na Channel (agonist, no delayed inactivation)	I1, M	III, XI
κ (KAPPA)	Voltage-gated K Channel (blocker)	A, I2, J, M, O1	III, IV, VI/VII, XI, XIV
μ (MU)	Voltage-gated Na Channel (antagonist, blocker)	M, O1,T	III, IV, V, VI, VII
ρ (RHO)	Alpha 1 adrenoreceptor (GPCR)	A	I
σ (SIGMA)	Serotonin-gated ion channels (GPCR)	S	VIII
τ (TAU)	Somatostatin receptor	T	V
χ (CHI)	Neuronal noradrenaline transporter	T	X
ω (OMEGA)	Voltage-gated calcium channel	O1, O2	VI/VII, XVI, XXVI

**Table 2:** Drug development with conotoxins.

	Conopeptide	Commercial Name	Comment	Target	Stage	Company	*Conus* Species (**)	Reference
1	α-Vc1.1	ACV1	Neuropathic pain	nAChR (α9α10)	Phase II *	Metabolic Pharmaceuticals, Melbourne, Australia	*Conus victoriae* (m)	[3]
2	ω-CVID	AM336	Neuropathic pain	Ca^2+^ channel (CaV2.2) N-type calcium channels/blocker	Phase IIa *	Relevare Pharmaceuticals LTD., Australia	*Conus catus* (p)	[3]
3	μO-MrVIB	CGX-1002	Neuropathic pain	Sodium channels/subtype selective blocker	Preclinical *	Cognetix Inc, Salt Lake City, USA	*Conus tulipa* (p)	[3]
4	Conantokin-G	CGX-1007	Intractable epilepsy / pain	NMDA receptor (NR2B)	Preclinical *	Cognetix Inc, Salt Lake City, USA	*Conus geographus* (p)	[3]
5	Contulakin-G	CGX-1160	Neuropathic pain	Neurotensin receptor	Phase Ib *	Cognetix Inc, Salt Lake City, USA	*Conus geographus* (p)	[3]
6	ω-MVIIA	SNX-III, C1002, Ziconotide, Prialt	Intractable pain	Ca^2+^ channel (CaV2.2) N-type calcium channels/blocker	FDA-approved	Elan Corporation (Elan Pharmaceuticals), CA, USA	*Conus magus* (p)	[3]
7	χ-MrIA	Xen2174	Neuropathic pain	Norepinephrine transporter/inhibitor	Phase IIa *	Xenome, Ltd., Brisbane, Qld., Australia	n.a.	[3]
8	κ-PVIIA	CGX-1051	Acute Myocardial Infarct, Cardioprotection	K^+^ channel (KV1)/blocker	Preclinical	n.a.	n.a.	[64]
9	n.a.	CGX-1204	Muscle relaxer / pain	Nicotinic acetylcholine receptors/antagonist	Preclinical	n.a.	n.a.	[64]
10	μ-SIIIA	PEG-SIIIA	Inflammatory pain	Sodium channels/blocker	Preclinical	n.a.	n.a.	[64]
11	ρ-Conotoxin TIA	n.a.	n.a.	α-1 adrenergic receptors	Preclinical	Xenome, Ltd., Brisbane, Qld., Australia	*Conus tulipa* (p)	[65]
12	χ-conopeptides (χ-CTX MrIA/B)	n.a.	Neuropathic pain	Neurotransmitter transporters	Preclinical	Xenome, Ltd., Brisbane, Qld., Australia	*Conus marmoreus* (m)	[65]

* indicates that further research has been known as terminated; ** Prey preference for *Conus* species: p = piscivorous (fish-hunting); m = molluscivorous (mollusk hunting); v = vermivorous (worm-hunting).

## Supplementary Materials

The following are available online at https://www.mdpi.com/article/10.3390/md20020105/s1, Table S1. Conidae list and their feeding habits information; Table S2. List of *Conus* species contained by the phylogenetic tree; Table S3. Raw data of the radar plot input table; Table S4. Input data for the radar plot.
