# Supplementary material for: Biomedical Potential of the Neglected Molluscivorous and Vermivorous Conus Species
Source: Mar Drugs. 2022 Jan 27;20(2):105. doi: 10.3390/md20020105 (PMC8878422; doi:10.3390/md20020105)
Supplement: Supplementary file 1 [file marinedrugs-20-00105-s001.zip › marinedrugs-1527294-supplementary.pdf]

Supplementary

Biomedical Potential of the Neglected Molluscivorous and Vermivorous *Conus* Species

Table S1 Conidae list and their feeding habits information

Number	If Present on the tree	Genus	Species	Feeding Habits	Other Remarks
1	N	*Conasprella*	*ageri*	†	
2	N	*Conasprella*	*aquitanica*	†	
3	N	*Conasprella*	*aturensis*	†	
4	N	*Conasprella*	*berwerthi*	†	
5	N	*Conasprella*	*burckhardti*	†	
6	N	*Conasprella*	*cercadensis*	†	
7	N	*Conasprella*	*imitator*	†	
8	N	*Conasprella*	*kitteredgei*	†	
9	N	*Conasprella*	*minutissima*	†	
10	N	*Conasprella*	*stenostoma*	†	
11	N	*Conasprella*	*subturrita*	†	
12	Y	*Conus*	*amadis*	Molluscivore	
13	Y	*Conus*	*ammiralis*	Molluscivore	
14	Y	*Conus*	*araneosus*	Molluscivore	
15	Y	*Conus*	*aulicus*	Molluscivore	
16	Y	*Conus*	*aureus*	Molluscivore	
17	Y	*Conus*	*auricomus*	Molluscivore	
18	Y	*Conus*	*bandanus*	Molluscivore	
19	Y	*Conus*	*bengalensis*	Molluscivore	
20	Y	*Conus*	*canonicus*	Molluscivore	
21	Y	*Conus*	*crocatus*	Molluscivore	
22	Y	*Conus*	*dalli*	Molluscivore	
23	Y	*Conus*	*episcopatus*	Molluscivore	
24	Y	*Conus*	*furvus*	Molluscivore	

25	Y	*Conus*	*gloriamaris*	Molluscivore
26	Y	*Conus*	*immelmani*	Molluscivore
27	Y	*Conus*	*jucundus*	Molluscivore
28	Y	*Conus*	*legatus*	Molluscivore
29	Y	*Conus*	*locumtenens*	Molluscivore
30	Y	*Conus*	*lohri*	Molluscivore
31	Y	*Conus*	*magnificus*	Molluscivore
32	Y	*Conus*	*marmoreus*	Molluscivore
33	Y	*Conus*	*natalis*	Molluscivore
34	Y	*Conus*	*omaria*	Molluscivore
35	Y	*Conus*	*pennaceus*	Molluscivore
36	Y	*Conus*	*retifer*	Molluscivore
37	Y	*Conus*	*textile*	Molluscivore
38	Y	*Conus*	*thalassiarchus*	Molluscivore
39	Y	*Conus*	*thomae*	Molluscivore
40	Y	*Conus*	*victoriae*	Molluscivore
41	N	*Conus*	*archiepiscopus*	Molluscivore
42	N	*Conus*	*praelatus*	Molluscivore
43	N	*Conus*	*purus*	Molluscivore
44	N	*Conus*	*abbas*	Molluscivore
45	N	*Conus*	*auratinus*	Molluscivore
46	N	*Conus*	*barbieri*	Molluscivore
47	N	*Conus*	*behelokensis*	Molluscivore
48	N	*Conus*	*cordigera*	Molluscivore
49	N	*Conus*	*echo*	Molluscivore
50	N	*Conus*	*glorioceanus*	Molluscivore
51	N	*Conus*	*hamanni*	Molluscivore
52	N	*Conus*	*kawamurai*	Molluscivore
53	N	*Conus*	*lamberti*	Molluscivore
54	N	*Conus*	*laueri*	Molluscivore
55	N	*Conus*	*madagascariensis*	Molluscivore
56	N	*Conus*	*marchionatus*	Molluscivore

57	N	*Conus*	*milneedwardsi*	Molluscivore	
58	N	*Conus*	*natalaurantius*	Molluscivore	
59	N	*Conus*	*nodulosus*	Molluscivore	
60	N	*Conus*	*scottjordani*	Molluscivore	
61	N	*Conus*	*tagaroae*	Molluscivore	
62	N	*Conus*	*telatus*	Molluscivore	
63	N	*Profundiconus*	*dondani*	Molluscivore	
64	N	*Profundiconus*	*pacificus*	Molluscivore	
65	Y	*Conus*	*dusaveli*	Molluscivore / Piscivore	
66	Y	*Conus*	*purpurascens*	Piscivore	earthworms
67	Y	*Conus*	*ermineus*	Piscivore	Mollusc/Octopus
68	N	*Conus*	*subulatus*	Piscivore	nomen dubium
69	Y	*Conus*	*achatinus*	Piscivore	
70	Y	*Conus*	*andamanensis*	Piscivore	
71	Y	*Conus*	*aurisiacus*	Piscivore	
72	Y	*Conus*	*barthelemyi*	Piscivore	
73	Y	*Conus*	*catus*	Piscivore	
74	Y	*Conus*	*cervus*	Piscivore	
75	Y	*Conus*	*cinereus*	Piscivore	
76	Y	*Conus*	*circumcisus*	Piscivore	
77	Y	*Conus*	*consors*	Piscivore	
78	Y	*Conus*	*cuvieri*	Piscivore	
79	Y	*Conus*	*floccatus*	Piscivore	
80	Y	*Conus*	*gauguini*	Piscivore	
81	Y	*Conus*	*geographus*	Piscivore	
82	Y	*Conus*	*grangeri*	Piscivore	
83	Y	*Conus*	*gubernator*	Piscivore	
84	Y	*Conus*	*koukae*	Piscivore	
85	Y	*Conus*	*magus*	Piscivore	
86	Y	*Conus*	*monachus*	Piscivore	
87	Y	*Conus*	*mucronatus*	Piscivore	
88	Y	*Conus*	*nigropunctatus*	Piscivore	
89	Y	*Conus*	*obscurus*	Piscivore	

90	Y	*Conus*	*parius*	Piscivore
91	Y	*Conus*	*proximus*	Piscivore
92	Y	*Conus*	*simonis*	Piscivore
93	Y	*Conus*	*stercusmuscarum*	Piscivore
94	Y	*Conus*	*striatus*	Piscivore
95	Y	*Conus*	*striolatus*	Piscivore
96	Y	*Conus*	*sutanorcum*	Piscivore
97	Y	*Conus*	*tulipa*	Piscivore
98	Y	*Profundiconus*	*kanakinus*	Piscivore
99	Y	*Profundiconus*	*profundorum*	Piscivore
100	N	*Conus*	*morrisoni*	Piscivore
101	N	*Conus*	*adamsonii*	Piscivore
102	N	*Conus*	*alabaster*	Piscivore
103	N	*Conus*	*alexandrei*	Piscivore
104	N	*Conus*	*angioiorum*	Piscivore
105	N	*Conus*	*arafurensis*	Piscivore
106	N	*Conus*	*asiaticus*	Piscivore
107	N	*Conus*	*balabacensis*	Piscivore
108	N	*Conus*	*barbara*	Piscivore
109	N	*Conus*	*boutetorum*	Piscivore
110	N	*Conus*	*cebuensis*	Piscivore
111	N	*Conus*	*chiapponorum*	Piscivore
112	N	*Conus*	*collisus*	Piscivore
113	N	*Conus*	*dampierensis*	Piscivore
114	N	*Conus*	*eldredi*	Piscivore
115	N	*Conus*	*fischoederi*	Piscivore
116	N	*Conus*	*fragilissimus*	Piscivore
117	N	*Conus*	*fulmen*	Piscivore
118	N	*Conus*	*gilvus*	Piscivore
119	N	*Conus*	*giorossii*	Piscivore
120	N	*Conus*	*goudeyi*	Piscivore
121	N	*Conus*	*habui*	Piscivore

122	N	*Conus*	*jickelii*	Piscivore	
123	N	*Conus*	*julii*	Piscivore	
124	N	*Conus*	*leobottonii*	Piscivore	
125	N	*Conus*	*leobrerai*	Piscivore	
126	N	*Conus*	*lizardensis*	Piscivore	
127	N	*Conus*	*marielae*	Piscivore	
128	N	*Conus*	*martinianus*	Piscivore	
129	N	*Conus*	*niederhoeferi*	Piscivore	
130	N	*Conus*	*robini*	Piscivore	
131	N	*Conus*	*rouxi*	Piscivore	
132	N	*Conus*	*salzmanni*	Piscivore	
133	N	*Conus*	*santinii*	Piscivore	
134	N	*Conus*	*sartii*	Piscivore	
135	N	*Conus*	*scalptus*	Piscivore	
136	N	*Conus*	*sculpturatus*	Piscivore	
137	N	*Conus*	*sertacinctus*	Piscivore	
138	N	*Conus*	*sogodensis*	Piscivore	
139	N	*Conus*	*solangeae*	Piscivore	
140	N	*Conus*	*solomonensis*	Piscivore	
141	N	*Conus*	*stramineus*	Piscivore	
142	N	*Conus*	*timorensis*	Piscivore	
143	N	*Conus*	*vappereaui*	Piscivore	
144	N	*Conus*	*vicweei*	Piscivore	
145	N	*Conus*	*yemenensis*	Piscivore	
146	N	*Conus*	*zandbergeni*	Piscivore	
147	N	*Conus*	*zapatosensis*	Piscivore	
148	N	*Conus*	*zebra*	Piscivore	
149	Y	*Conus*	*bullatus*	Piscivore / Molluscivore	
150	Y	*Conasprella*	*aphrodite*	Vermivore	
151	Y	*Conasprella*	*arcuata*	Vermivore	
152	Y	*Conasprella*	*baileyi*	Vermivore	
153	Y	*Conasprella*	*comatosa*	Vermivore	

154	Y	*Conasprella*	*delessertii*	Vermivore
155	Y	*Conasprella*	*eugrammata*	Vermivore
156	Y	*Conasprella*	*guidopoppei*	Vermivore
157	Y	*Conasprella*	*hopwoodi*	Vermivore
158	Y	*Conasprella*	*ichinoseana*	Vermivore
159	Y	*Conasprella*	*kimioi*	Vermivore
160	Y	*Conasprella*	*longurionis*	Vermivore
161	Y	*Conasprella*	*mazei*	Vermivore
162	Y	*Conasprella*	*memiae*	Vermivore
163	Y	*Conasprella*	*mindana*	Vermivore
164	Y	*Conasprella*	*orbignyi*	Vermivore
165	Y	*Conasprella*	*otohimeae*	Vermivore
166	Y	*Conasprella*	*pagoda*	Vermivore
167	Y	*Conasprella*	*perplexa*	Vermivore
168	Y	*Conasprella*	*pseudokimioi*	Vermivore
169	Y	*Conasprella*	*puncticulata*	Vermivore
170	Y	*Conasprella*	*stearnsii*	Vermivore
171	Y	*Conasprella*	*tornata*	Vermivore
172	Y	*Conasprella*	*viminea*	Vermivore
173	Y	*Conasprella*	*wakayamaensis*	Vermivore
174	Y	*Conus*	*abbreviatus*	Vermivore
175	Y	*Conus*	*acutangulus*	Vermivore
176	Y	*Conus*	*alconnelli*	Vermivore
177	Y	*Conus*	*amphiurgus*	Vermivore
178	Y	*Conus*	*anabathrum*	Vermivore
179	Y	*Conus*	*anemone*	Vermivore
180	Y	*Conus*	*angasi*	Vermivore
181	Y	*Conus*	*antoniaensis*	Vermivore
182	Y	*Conus*	*antoniomonteiroi*	Vermivore
183	Y	*Conus*	*arangoi*	Vermivore
184	Y	*Conus*	*archon*	Vermivore
185	Y	*Conus*	*ardisiaceus*	Vermivore

186	Y	*Conus*	*arenatus*	Vermivore	
187	Y	*Conus*	*aristophanes*	Vermivore	
188	Y	*Conus*	*ateralbus*	Vermivore	
189	Y	*Conus*	*augur*	Vermivore	
190	Y	*Conus*	*australis*	Vermivore	
191	Y	*Conus*	*balteatus*	Vermivore	
192	Y	*Conus*	*bartschi*	Vermivore	
193	Y	*Conus*	*belairensis*	Vermivore	
194	Y	*Conus*	*betulinus*	Vermivore	
195	Y	*Conus*	*biliosus*	Vermivore	
196	Y	*Conus*	*boavistensis*	Vermivore	
197	Y	*Conus*	*boeticus*	Vermivore	
198	Y	*Conus*	*borgesi*	Vermivore	
199	Y	*Conus*	*bruguieresi*	Vermivore	
200	Y	*Conus*	*brunneus*	Vermivore	
201	Y	*Conus*	*buxeus*	Vermivore	
202	Y	*Conus*	*buxeus loroisii*	Vermivore	
203	Y	*Conus*	*byssinus*	Vermivore	
204	Y	*Conus*	*calhetae*	Vermivore	
205	Y	*Conus*	*cancellatus*	Vermivore	
206	Y	*Conus*	*capitanellus*	Vermivore	
207	Y	*Conus*	*capitaneus*	Vermivore	
208	Y	*Conus*	*caracteristicus*	Vermivore	
209	Y	*Conus*	*cedonulli*	Vermivore	
210	Y	*Conus*	*chaldaeus*	Vermivore	
211	Y	*Conus*	*chiangi*	Vermivore	
212	Y	*Conus*	*cloveri*	Vermivore	
213	Y	*Conus*	*coelinae*	Vermivore	
214	Y	*Conus*	*coffeae*	Vermivore	
215	Y	*Conus*	*corallinus*	Vermivore	
216	Y	*Conus*	*coronatus*	Vermivore	
217	Y	*Conus*	*crotchii*	Vermivore	

218	Y	*Conus*	*cuneolus*	Vermivore
219	Y	*Conus*	*curassaviensis*	Vermivore
220	Y	*Conus*	*curralensis*	Vermivore
221	Y	*Conus*	*damottai*	Vermivore
222	Y	*Conus*	*daucus*	Vermivore
223	Y	*Conus*	*dayriti*	Vermivore
224	Y	*Conus*	*decoratus*	Vermivore
225	Y	*Conus*	*delanoyae*	Vermivore
226	Y	*Conus*	*denizi*	Vermivore
227	Y	*Conus*	*diadema*	Vermivore
228	Y	*Conus*	*diminutus*	Vermivore
229	Y	*Conus*	*distans*	Vermivore
230	Y	*Conus*	*dorotheae*	Vermivore
231	Y	*Conus*	*dorreensis*	Vermivore
232	Y	*Conus*	*ebraeus*	Vermivore
233	Y	*Conus*	*echinophilus*	Vermivore
234	Y	*Conus*	*emaciatus*	Vermivore
235	Y	*Conus*	*excelsus*	Vermivore
236	Y	*Conus*	*eximius*	Vermivore
237	Y	*Conus*	*felitae*	Vermivore
238	Y	*Conus*	*fergusoni*	Vermivore
239	Y	*Conus*	*fernandesi*	Vermivore
240	Y	*Conus*	*ferrugineus*	Vermivore
241	Y	*Conus*	*figulinus*	Vermivore
242	Y	*Conus*	*flavescens*	Vermivore
243	Y	*Conus*	*flavidus*	Vermivore
244	Y	*Conus*	*flavus*	Vermivore
245	Y	*Conus*	*floridulus*	Vermivore
246	Y	*Conus*	*frigidus*	Vermivore
247	Y	*Conus*	*fuscoflavus*	Vermivore
248	Y	*Conus*	*genuanus*	Vermivore
249	Y	*Conus*	*gladiator*	Vermivore

250	Y	*Conus*	*glans*	Vermivore	
251	Y	*Conus*	*gondwanensis*	Vermivore	
252	Y	*Conus*	*gonsaloi*	Vermivore	
253	Y	*Conus*	*gradatus*	Vermivore	
254	Y	*Conus*	*grahami*	Vermivore	
255	Y	*Conus*	*granum*	Vermivore	
256	Y	*Conus*	*guanche*	Vermivore	
257	Y	*Conus*	*guinaicus*	Vermivore	
258	Y	*Conus*	*hieroglyphus*	Vermivore	
259	Y	*Conus*	*hirasei*	Vermivore	
260	Y	*Conus*	*imperialis*	Vermivore	
261	Y	*Conus*	*infinitus*	Vermivore	
262	Y	*Conus*	*infrenatus*	Vermivore	
263	Y	*Conus*	*inscriptus*	Vermivore	
264	Y	*Conus*	*isabelarum*	Vermivore	
265	Y	*Conus*	*janus*	Vermivore	
266	Y	*Conus*	*josephinae*	Vermivore	
267	Y	*Conus*	*judaeus*	Vermivore	
268	Y	*Conus*	*kintoki*	Vermivore	
269	Y	*Conus*	*klemae*	Vermivore	
270	Y	*Conus*	*lenavati*	Vermivore	
271	Y	*Conus*	*leopardus*	Vermivore	
272	Y	*Conus*	*lischkeanus*	Vermivore	
273	Y	*Conus*	*litoglyphus*	Vermivore	
274	Y	*Conus*	*litteratus*	Vermivore	
275	Y	*Conus*	*lividus*	Vermivore	
276	Y	*Conus*	*longilineus*	Vermivore	
277	Y	*Conus*	*lozeti*	Vermivore	
278	Y	*Conus*	*lugubris*	Vermivore	
279	Y	*Conus*	*luteus*	Vermivore	
280	Y	*Conus*	*lynceus*	Vermivore	
281	Y	*Conus*	*madecassinus*	Vermivore	

282	Y	*Conus*	*maioensis*	Vermivore
283	Y	*Conus*	*medoci*	Vermivore
284	Y	*Conus*	*melvilli*	Vermivore
285	Y	*Conus*	*mercator*	Vermivore
286	Y	*Conus*	*miles*	Vermivore
287	Y	*Conus*	*miliaris*	Vermivore
288	Y	*Conus*	*miruchae*	Vermivore
289	Y	*Conus*	*mitratus*	Vermivore
290	Y	*Conus*	*monile*	Vermivore
291	Y	*Conus*	*moreleti*	Vermivore
292	Y	*Conus*	*mozambicus*	Vermivore
293	Y	*Conus*	*muriculatus*	Vermivore
294	Y	*Conus*	*mus*	Vermivore
295	Y	*Conus*	*musicus*	Vermivore
296	Y	*Conus*	*mustelinus*	Vermivore
297	Y	*Conus*	*namocanus*	Vermivore
298	Y	*Conus*	*navarroi*	Vermivore
299	Y	*Conus*	*neptunus*	Vermivore
300	Y	*Conus*	*nucleus*	Vermivore
301	Y	*Conus*	*nux*	Vermivore
302	Y	*Conus*	*ochroleucus*	Vermivore
303	Y	*Conus*	*orion*	Vermivore
304	Y	*Conus*	*parvatus*	Vermivore
305	Y	*Conus*	*patricius*	Vermivore
306	Y	*Conus*	*pertusus*	Vermivore
307	Y	*Conus*	*philippii*	Vermivore
308	Y	*Conus*	*pictus*	Vermivore
309	Y	*Conus*	*planorbis*	Vermivore
310	Y	*Conus*	*plinthis*	Vermivore
311	Y	*Conus*	*poormani*	Vermivore
312	Y	*Conus*	*praecellens*	Vermivore
313	Y	*Conus*	*princeps*	Vermivore

314	Y	*Conus*	*pulcher*	Vermivore	
315	Y	*Conus*	*pulicarius*	Vermivore	
316	Y	*Conus*	*queenslandis*	Vermivore	
317	Y	*Conus*	*quercinus*	Vermivore	
318	Y	*Conus*	*rattus*	Vermivore	
319	Y	*Conus*	*raulsilvai*	Vermivore	
320	Y	*Conus*	*recurvus*	Vermivore	
321	Y	*Conus*	*regius*	Vermivore	
322	Y	*Conus*	*regonae*	Vermivore	
323	Y	*Conus*	*richeri*	Vermivore	
324	Y	*Conus*	*roeckeli*	Vermivore	
325	Y	*Conus*	*roseorapum*	Vermivore	
326	Y	*Conus*	*sandwichensis*	Vermivore	
327	Y	*Conus*	*sanguinolentus*	Vermivore	
328	Y	*Conus*	*shikamai*	Vermivore	
329	Y	*Conus*	*spectrum*	Vermivore	
330	Y	*Conus*	*sponsalis*	Vermivore	
331	Y	*Conus*	*spurius*	Vermivore	
332	Y	*Conus*	*striatellus*	Vermivore	
333	Y	*Conus*	*sugimotonis*	Vermivore	
334	Y	*Conus*	*sulcatus*	Vermivore	
335	Y	*Conus*	*suturatus*	Vermivore	
336	Y	*Conus*	*tabidus*	Vermivore	
337	Y	*Conus*	*taeniatus*	Vermivore	
338	Y	*Conus*	*tenuistriatus*	Vermivore	
339	Y	*Conus*	*terebra*	Vermivore	
340	Y	*Conus*	*tinianus*	Vermivore	
341	Y	*Conus*	*tribblei*	Vermivore	
342	Y	*Conus*	*trochulus*	Vermivore	
343	Y	*Conus*	*unifasciatus*	Vermivore	
344	Y	*Conus*	*varius*	Vermivore	
345	Y	*Conus*	*ventricosus*	Vermivore	

346	Y	*Conus*	*venulatus*	Vermivore	
347	Y	*Conus*	*verdensis*	Vermivore	
348	Y	*Conus*	*vexillum*	Vermivore	
349	Y	*Conus*	*villepinii*	Vermivore	
350	Y	*Conus*	*viola*	Vermivore	
351	Y	*Conus*	*violaceus*	Vermivore	
352	Y	*Conus*	*virgatus*	Vermivore	
353	Y	*Conus*	*virgo*	Vermivore	
354	Y	*Conus*	*vittatus*	Vermivore	
355	Y	*Conus*	*vitulinus*	Vermivore	
356	Y	*Conus*	*voluminalis*	Vermivore	
357	Y	*Conus*	*vulcanus*	Vermivore	
358	Y	*Conus*	*xicoi*	Vermivore	
359	Y	*Conus*	*zeylanicus*	Vermivore	
360	Y	*Conus*	*zonatus*	Vermivore	
361	Y	*Profundiconus*	*vaubani*	Vermivore	
362	Y	*Conus*	*eversoni*	Vermivore	
363	Y	*Conus*	*generalis*	Vermivore	
364	Y	*Conus*	*martensi*	Vermivore	
365	N	*Conasprella*	*jaspideus*	Vermivore	
366	N	*Conasprella*	*hivana*	Vermivore	
367	N	*Conasprella*	*lentiginosa*	Vermivore	
368	N	*Conasprella*	*traversiana*	Vermivore	
369	N	*Conus*	*abrolhosensis*	Vermivore	
370	N	*Conus*	*advertex*	Vermivore	
371	N	*Conus*	*aemulus*	Vermivore	
372	N	*Conus*	*africanus*	Vermivore	
373	N	*Conus*	*alainallaryi*	Vermivore	
374	N	*Conus*	*albuquerquei*	Vermivore	
375	N	*Conus*	*alexandrinus*	Vermivore	
376	N	*Conus*	*aplustre*	Vermivore	
377	N	*Conus*	*armadillo*	Vermivore	

378	N	*Conus*	*artoptus*	Vermivore	
379	N	*Conus*	*aureonimbosus*	Vermivore	
380	N	*Conus*	*austroviola*	Vermivore	
381	N	*Conus*	*baeri*	Vermivore	
382	N	*Conus*	*bahamensis*	Vermivore	
383	N	*Conus*	*bayani*	Vermivore	
384	N	*Conus*	*bayeri*	Vermivore	
385	N	*Conus*	*belizeanus*	Vermivore	
386	N	*Conus*	*bellocqae*	Vermivore	
387	N	*Conus*	*bellulus*	Vermivore	
388	N	*Conus*	*bessei*	Vermivore	
389	N	*Conus*	*binghamae*	Vermivore	
390	N	*Conus*	*bonfigliolii*	Vermivore	
391	N	*Conus*	*boui*	Vermivore	
392	N	*Conus*	*broderipii*	Vermivore	
393	N	*Conus*	*brunneobandatus*	Vermivore	
394	N	*Conus*	*burryae*	Vermivore	
395	N	*Conus*	*caillaudii*	Vermivore	
396	N	*Conus*	*capreolus*	Vermivore	
397	N	*Conus*	*carcellesi*	Vermivore	
398	N	*Conus*	*clarus*	Vermivore	
399	N	*Conus*	*clerii*	Vermivore	
400	N	*Conus*	*cocceus*	Vermivore	
401	N	*Conus*	*colmani*	Vermivore	
402	N	*Conus*	*colombi*	Vermivore	
403	N	*Conus*	*coltrorum*	Vermivore	
404	N	*Conus*	*compressus*	Vermivore	
405	N	*Conus*	*conspersus*	Vermivore	
406	N	*Conus*	*cuna*	Vermivore	
407	N	*Conus*	*cyanostoma*	Vermivore	
408	N	*Conus*	*cylindraceus*	Vermivore	
409	N	*Conus*	*desidiosus*	Vermivore	

410	N	*Conus*	*edaphus*	Vermivore
411	N	*Conus*	*escondidai*	Vermivore
412	N	*Conus*	*estivali*	Vermivore
413	N	*Conus*	*felix*	Vermivore
414	N	*Conus*	*fijisulcatus*	Vermivore
415	N	*Conus*	*flammeacolor*	Vermivore
416	N	*Conus*	*gabelishi*	Vermivore
417	N	*Conus*	*garywilsoni*	Vermivore
418	N	*Conus*	*gigasulcatus*	Vermivore
419	N	*Conus*	*glaucus*	Vermivore
420	N	*Conus*	*glenni*	Vermivore
421	N	*Conus*	*glicksteini*	Vermivore
422	N	*Conus*	*gloriakiiensis*	Vermivore
423	N	*Conus*	*goajira*	Vermivore
424	N	*Conus*	*hazinorum*	Vermivore
425	N	*Conus*	*helgae*	Vermivore
426	N	*Conus*	*hennequini*	Vermivore
427	N	*Conus*	*honkeri*	Vermivore
428	N	*Conus*	*hyaena*	Vermivore
429	N	*Conus*	*hyaena concolor*	Vermivore
430	N	*Conus*	*iodostoma*	Vermivore
431	N	*Conus*	*jorioi*	Vermivore
432	N	*Conus*	*kaiserae*	Vermivore
433	N	*Conus*	*kermadecensis*	Vermivore
434	N	*Conus*	*kersteni*	Vermivore
435	N	*Conus*	*kevani*	Vermivore
436	N	*Conus*	*kremerorum*	Vermivore
437	N	*Conus*	*kuroharai*	Vermivore
438	N	*Conus*	*leekremeri*	Vermivore
439	N	*Conus*	*lemniscatus*	Vermivore
440	N	*Conus*	*levistimpsoni*	Vermivore
441	N	*Conus*	*lienardi*	Vermivore

442	N	*Conus*	*lightbourni*	Vermivore
443	N	*Conus*	*limpusi*	Vermivore
444	N	*Conus*	*lindae*	Vermivore
445	N	*Conus*	*malacanus*	Vermivore
446	N	*Conus*	*maya*	Vermivore
447	N	*Conus*	*mcbridei*	Vermivore
448	N	*Conus*	*nanus*	Vermivore
449	N	*Conus*	*nielsenae*	Vermivore
450	N	*Conus*	*oishii*	Vermivore
451	N	*Conus*	*papilliferus*	Vermivore
452	N	*Conus*	*papuensis*	Vermivore
453	N	*Conus*	*parascalaris*	Vermivore
454	N	*Conus*	*patae*	Vermivore
455	N	*Conus*	*patriceae*	Vermivore
456	N	*Conus*	*paukstisi*	Vermivore
457	N	*Conus*	*peasei*	Vermivore
458	N	*Conus*	*penchaszadehi*	Vermivore
459	N	*Conus*	*petergabrieli*	Vermivore
460	N	*Conus*	*portobeloensis*	Vermivore
461	N	*Conus*	*poulosi*	Vermivore
462	N	*Conus*	*pretiosus*	Vermivore
463	N	*Conus*	*primus*	Vermivore
464	N	*Conus*	*quiquandoni*	Vermivore
465	N	*Conus*	*ranonganus*	Vermivore
466	N	*Conus*	*recluzianus*	Vermivore
467	N	*Conus*	*reductaspiralis*	Vermivore
468	N	*Conus*	*ritae*	Vermivore
469	N	*Conus*	*rufimaculosus*	Vermivore
470	N	*Conus*	*salletae*	Vermivore
471	N	*Conus*	*santanaensis*	Vermivore
472	N	*Conus*	*saragasae*	Vermivore
473	N	*Conus*	*shaskyi*	Vermivore

474	N	*Conus*	*stimpsoni*	Vermivore	
475	N	*Conus*	*sulcocastaneus*	Vermivore	
476	N	*Conus*	*suratensis*	Vermivore	
477	N	*Conus*	*swainsoni*	Vermivore	
478	N	*Conus*	*tacomae*	Vermivore	
479	N	*Conus*	*terryni*	Vermivore	
480	N	*Conus*	*therriaulti*	Vermivore	
481	N	*Conus*	*tisii*	Vermivore	
482	N	*Conus*	*tostesi*	Vermivore	
483	N	*Conus*	*trencarti*	Vermivore	
484	N	*Conus*	*trigonus*	Vermivore	
485	N	*Conus*	*typhon*	Vermivore	
486	N	*Conus*	*umbelinae*	Vermivore	
487	N	*Conus*	*vayssierei*	Vermivore	
488	N	*Conus*	*venezuelanus*	Vermivore	
489	N	*Conus*	*vikingorum*	Vermivore	
490	N	*Conus*	*visagenus*	Vermivore	
491	N	*Conus*	*wallangra*	Vermivore	
492	N	*Conus*	*wandae*	Vermivore	
493	N	*Conus*	*wittigi*	Vermivore	
494	N	*Conus*	*xanthicus*	Vermivore	
495	N	*Conus*	*xanthocinctus*	Vermivore	
496	N	*Profundiconus*	*teramachii*	Vermivore	
497	Y	*Conus*	*mappa*	Vermivore / Molluscivore	
498	Y	*Conus*	*nobilis*	Vermivore / Molluscivore	
499	Y	*Conus*	*nussatella*	Vermivore / Molluscivore	
500	N	*Conus*	*ambiguus*	Vermivore / Molluscivore	
501	N	*Conus*	*royaikeni*	Vermivore / Molluscivore	
502	N	*Conus*	*sydneyensis*	Vermivore / Molluscivore	
503	Y	*Conus*	*blanfordianus*	Vermivore / Piscivore	
504	Y	*Conus*	*bruuni*	Vermivore / Piscivore	
505	Y	*Conus*	*eburneus*	Vermivore / Piscivore	

506	Y	*Conus*	*kinoshitai*	Vermivore / Piscivore	
507	Y	*Conus*	*laterculatus*	Vermivore / Piscivore	
508	Y	*Conus*	*moluccensis*	Vermivore / Piscivore	
509	Y	*Conus*	*pergrandis*	Vermivore / Piscivore	
510	Y	*Conus*	*radiatus*	Vermivore / Piscivore	
511	Y	*Conus*	*rolani*	Vermivore / Piscivore	
512	Y	*Conus*	*tessulatus*	Vermivore / Piscivore	
513	N	*Conus*	*ciderryi*	Vermivore / Piscivore	
514	N	*Conus*	*erythraeensis*	Vermivore / Piscivore	
515	N	*Conus*	*granulatus*	Vermivore / Piscivore	
516	N	*Conus*	*kiicumulus*	Vermivore / Piscivore	
517	N	*Conus*	*nigromaculatus*	Vermivore / Piscivore	
518	Y	*Californiconus*	*californicus*	VMP	
519	N	*Conus*	*alatus*		nomen dubium
520	N	*Conus*	*albidus*		nomen dubium
521	N	*Conus*	*amethysteus*		nomen dubium
522	Y	*Conus*	*biliosus parvulus*		
523	Y	*Conasprella*	*alisi*		
524	Y	*Conasprella*	*boholensis*		
525	Y	*Conasprella*	*boucheti*		
526	Y	*Conasprella*	*coriolisi*		
527	Y	*Conasprella*	*elokismenos*		
528	Y	*Conasprella*	*ione*		
529	Y	*Conasprella*	*joliveti*		
530	Y	*Conasprella*	*mahogani*		
531	Y	*Conasprella*	*pseudorbignyi*		
532	Y	*Conasprella*	*sieboldii*		
533	Y	*Conasprella*	*ximenes*		
534	Y	*Conus*	*andremenezi*		
535	Y	*Conus*	*chytreus*		
536	Y	*Conus*	*circumactus*		
537	Y	*Conus*	*franciscoi*		

538	Y	*Conus*	*fumigatus*
539	Y	*Conus*	*hamamotoi*
540	Y	*Conus*	*jacarusoi*
541	Y	*Conus*	*jourdani*
542	Y	*Conus*	*lobitensis*
543	Y	*Conus*	*luciae*
544	Y	*Conus*	*micropunctatus*
545	Y	*Conus*	*miniexcelsus*
546	Y	*Conus*	*nimbosus*
547	Y	*Conus*	*pseudimperialis*
548	Y	*Conus*	*pseudonivifer*
549	Y	*Conus*	*richardbinghami*
550	Y	*Conus*	*stupa*
551	Y	*Conus*	*trovaoi*
552	Y	*Conus*	*variegatus*
553	Y	*Conus*	*zebroides*
554	Y	*Conus*	*zylmanae*
555	Y	*Profundiconus*	*smirna*
556	Y	*Conasprella*	*articulata*
557	Y	*Conasprella*	*centurio*
558	Y	*Conasprella*	*eucoronata*
559	Y	*Conasprella*	*lucida*
560	Y	*Conus*	*freitasi*
561	Y	*Conus*	*tiaratus*
562	Y	*Conus*	*franciscanus*
563	Y	*Conus*	*hughmorrisoni*
564	N	*Conus*	*aliwalensis*
565	N	*Conus*	*brandonensis*
566	N	*Conus*	*bratcherae*
567	N	*Conus*	*cathyae*
568	N	*Conus*	*chindeensis*
569	N	*Conus*	*corbieri*

570	N	*Conus*	*cymbioides*
571	N	*Conus*	*easoni*
572	N	*Conus*	*equiminaensis*
573	N	*Conus*	*estellae*
574	N	*Conus*	*eusebioi*
575	N	*Conus*	*fuscatus*
576	N	*Conus*	*galeyi*
577	N	*Conus*	*karubenthos*
578	N	*Conus*	*leehmani*
579	N	*Conus*	*maculospira*
580	N	*Conus*	*marimaris*
581	N	*Conus*	*markpagei*
582	N	*Conus*	*marysae*
583	N	*Conus*	*mascarenensis*
584	N	*Conus*	*michelcharlesi*
585	N	*Conus*	*mosterti*
586	N	*Conus*	*norpothi*
587	N	*Conus*	*nunesi*
588	N	*Conus*	*paulkersteni*
589	N	*Conus*	*philquiquandoni*
590	N	*Conus*	*pongo*
591	N	*Conus*	*potiguar*
592	N	*Conus*	*pseudocedonulli*
593	N	*Conus*	*quasidaucus*
594	N	*Conus*	*sanderi*
595	N	*Conus*	*scabriusculus*
596	N	*Conus*	*severinae*
597	N	*Conus*	*sugillatus*
598	N	*Conus*	*taitensis*
599	N	*Conus*	*tenorioi*
600	N	*Conus*	*thailandis*
601	N	*Conus*	*vezoi*

602	N	*Conasprella*	*tirardi*
603	N	*Conus*	*albellus*
604	N	*Conus*	*algoensis*
605	N	*Conus*	*allaryi*
606	N	*Conus*	*anabelae*
607	N	*Conus*	*athenae*
608	N	*Conus*	*attenuatus*
609	N	*Conus*	*aurantius*
610	N	*Conus*	*axelrodi*
611	N	*Conus*	*babaensis*
612	N	*Conus*	*bairstowi*
613	N	*Conus*	*beatrix*
614	N	*Conus*	*berdulinus*
615	N	*Conus*	*bocagei*
616	N	*Conus*	*bondarevi*
617	N	*Conus*	*brianhayesi*
618	N	*Conus*	*bulbus*
619	N	*Conus*	*cardinalis*
620	N	*Conus*	*carnalis*
621	N	*Conus*	*caysalensis*
622	N	*Conus*	*cepasi*
623	N	*Conus*	*coccineus*
624	N	*Conus*	*crosnieri*
625	N	*Conus*	*cumingii*
626	N	*Conus*	*danilai*
627	N	*Conus*	*dedonderi*
628	N	*Conus*	*deynzerorum*
629	N	*Conus*	*dianthus*
630	N	*Conus*	*dominicanus*
631	N	*Conus*	*donnae*
632	N	*Conus*	*duffyi*
633	N	*Conus*	*edwardpauli*

634	N	*Conus*	*eleutheraensis*
635	N	*Conus*	*empressae*
636	N	*Conus*	*encaustus*
637	N	*Conus*	*exiguus*
638	N	*Conus*	*explorator*
639	N	*Conus*	*filmeri*
640	N	*Conus*	*flavusalbus*
641	N	*Conus*	*fuscolineatus*
642	N	*Conus*	*gradatulus*
643	N	*Conus*	*granarius*
644	N	*Conus*	*gratacapii*
645	N	*Conus*	*hanshassi*
646	N	*Conus*	*harasewychi*
647	N	*Conus*	*harlandi*
648	N	*Conus*	*havanensis*
649	N	*Conus*	*ignotus*
650	N	*Conus*	*inconstans*
651	N	*Conus*	*inesae*
652	N	*Conus*	*julieandreae*
653	N	*Conus*	*kalafuti*
654	N	*Conus*	*kirkandersi*
655	N	*Conus*	*kulkulcan*
656	N	*Conus*	*lecourtorum*
657	N	*Conus*	*levis*
658	N	*Conus*	*lineopunctatus*
659	N	*Conus*	*lucaya*
660	N	*Conus*	*maculiferus*
661	N	*Conus*	*magellanicus*
662	N	*Conus*	*magnottei*
663	N	*Conus*	*maldivus*
664	N	*Conus*	*marileeae*
665	N	*Conus*	*medvedevi*

666	N	*Conus*	*milesi*
667	N	*Conus*	*minnamurra*
668	N	*Conus*	*monicae*
669	N	*Conus*	*montillai*
670	N	*Conus*	*naranjus*
671	N	*Conus*	*negroides*
672	N	*Conus*	*nobrei*
673	N	*Conus*	*ortneri*
674	N	*Conus*	*peli*
675	N	*Conus*	*petuchi*
676	N	*Conus*	*polongimarumai*
677	N	*Conus*	*pseudaurantius*
678	N	*Conus*	*pseudocardinalis*
679	N	*Conus*	*rawaiensis*
680	N	*Conus*	*rizali*
681	N	*Conus*	*rosalindensis*
682	N	*Conus*	*rosi*
683	N	*Conus*	*sahlbergi*
684	N	*Conus*	*sanguineus*
685	N	*Conus*	*scalarispira*
686	N	*Conus*	*scopulorum*
687	N	*Conus*	*solidus*
688	N	*Conus*	*sphacelatus*
689	N	*Conus*	*spiceri*
690	N	*Conus*	*splendidulus*
691	N	*Conus*	*stanfieldi*
692	N	*Conus*	*stupella*
693	N	*Conus*	*suduirauti*
694	N	*Conus*	*sukhadwalai*
695	N	*Conus*	*tenuilineatus*
696	N	*Conus*	*tethys*
697	N	*Conus*	*theodorei*

698	N	*Conus*	*troendlei*
699	N	*Conus*	*tuticorinensis*
700	N	*Conus*	*vautieri*
701	N	*Conus*	*velaensis*
702	N	*Conus*	*wilsi*
703	N	*Conus*	*ziczac*
704	N	*Conasprella*	*aculeiformis*
705	N	*Conasprella*	*acutimarginata*
706	N	*Conasprella*	*agassizi*
707	N	*Conasprella*	*alexandremonteiroi*
708	N	*Conasprella*	*allamandi*
709	N	*Conasprella*	*anacarolinae*
710	N	*Conasprella*	*anaglyptica*
711	N	*Conasprella*	*arawak*
712	N	*Conasprella*	*armiger*
713	N	*Conasprella*	*baccata*
714	N	*Conasprella*	*bajanensis*
715	N	*Conasprella*	*bermudensis*
716	N	*Conasprella*	*berschaueri*
717	N	*Conasprella*	*bodarti*
718	N	*Conasprella*	*booti*
719	N	*Conasprella*	*boriqua*
720	N	*Conasprella*	*bozzettii*
721	N	*Conasprella*	*branhamae*
722	N	*Conasprella*	*carlagrezziae*
723	N	*Conasprella*	*carvalhoi*
724	N	*Conasprella*	*chaac*
725	N	*Conasprella*	*chinchorroensis*
726	N	*Conasprella*	*coromandelica*
727	N	*Conasprella*	*crabosi*
728	N	*Conasprella*	*culebrana*
729	N	*Conasprella*	*damasoi*

730	N	*Conasprella*	*damasomonteiroi*		
731	N	*Conasprella*	*delucai*		
732	N	*Conasprella*	*dictator*		
733	N	*Conasprella*	*dieteri*		
734	N	*Conasprella*	*edpetuchi*		
735	N	*Conasprella*	*elegans*		
736	N	*Conasprella*	*emarginata*		
737	N	*Conasprella*	*ericmonnieri*		
738	N	*Conasprella*	*exumaensis*		
739	N	*Conasprella*	*fenzani*		
740	N	*Conasprella*	*ferreirai*		
741	N	*Conasprella*	*fijiensis*		
742	N	*Conasprella*	*fluviamaris*		
743	N	*Conasprella*	*gattegnoi*		
744	N	*Conasprella*	*geeraertsi*		
745	N	*Conasprella*	*gordyi*		
746	N	*Conasprella*	*grohi*		
747	N	*Conasprella*	*henckesi*		
748	N	*Conasprella*	*herndli*		
749	N	*Conasprella*	*howelli*		
750	N	*Conasprella*	*iansa*		
751	N	*Conasprella*	*icapui*		
752	N	*Conasprella*	*insculpta*		
753	N	*Conasprella*	*itapua*		
754	N	*Conasprella*	*ixchel*		
755	N	*Conasprella*	*janapatriceae*		
756	N	*Conasprella*	*janowskyae*		
757	N	*Conasprella*	*jaspidea*		
758	N	*Conasprella*	*joanae*		
759	N	*Conasprella*	*josei*		
760	N	*Conasprella*	*kantangana*		
761	N	*Conasprella*	*kellyae*		

762	N	*Conasprella*	*keppensi*
763	N	*Conasprella*	*kohni*
764	N	*Conasprella*	*lapulapui*
765	N	*Conasprella*	*lemuriana*
766	N	*Conasprella*	*lenhilli*
767	N	*Conasprella*	*levenensis*
768	N	*Conasprella*	*lindapowersae*
769	N	*Conasprella*	*lizarum*
770	N	*Conasprella*	*lorenzi*
771	N	*Conasprella*	*lusca*
772	N	*Conasprella*	*mackintoshi*
773	N	*Conasprella*	*marcusi*
774	N	*Conasprella*	*marinae*
775	N	*Conasprella*	*masinoi*
776	N	*Conasprella*	*mcgintyi*
777	N	*Conasprella*	*nereis*
778	N	*Conasprella*	*nodifera*
779	N	*Conasprella*	*ogum*
780	N	*Conasprella*	*olangoensis*
781	N	*Conasprella*	*oleiniki*
782	N	*Conasprella*	*pacei*
783	N	*Conasprella*	*paumotu*
784	N	*Conasprella*	*pealii*
785	N	*Conasprella*	*pepeiu*
786	N	*Conasprella*	*pfluegeri*
787	N	*Conasprella*	*philippequiquandoni*
788	N	*Conasprella*	*pomponeti*
789	N	*Conasprella*	*poremskii*
790	N	*Conasprella*	*prugnaudorum*
791	N	*Conasprella*	*pusio*
792	N	*Conasprella*	*rachelae*
793	N	*Conasprella*	*rainesae*

794	N	*Conasprella*	*ramalhoi*
795	N	*Conasprella*	*ramosorum*
796	N	*Conasprella*	*raoulensis*
797	N	*Conasprella*	*roatanensis*
798	N	*Conasprella*	*roberti*
799	N	*Conasprella*	*rutila*
800	N	*Conasprella*	*saecularis*
801	N	*Conasprella*	*sargenti*
802	N	*Conasprella*	*sauros*
803	N	*Conasprella*	*scaripha*
804	N	*Conasprella*	*schirrmeisteri*
805	N	*Conasprella*	*serafimi*
806	N	*Conasprella*	*simonei*
807	N	*Conasprella*	*somalica*
808	N	*Conasprella*	*stocki*
809	N	*Conasprella*	*tammymyersae*
810	N	*Conasprella*	*tayrona*
811	N	*Conasprella*	*tiki*
812	N	*Conasprella*	*toincabrali*
813	N	*Conasprella*	*torensis*
814	N	*Conasprella*	*traceyi*
815	N	*Conasprella*	*valianti*
816	N	*Conasprella*	*vanhyningi*
817	N	*Conasprella*	*vantwoudti*
818	N	*Conasprella*	*verrucosa*
819	N	*Conasprella*	*wendrosi*
820	N	*Conus*	*adami*
821	N	*Conus*	*adenensis*
822	N	*Conus*	*admirationis*
823	N	*Conus*	*aequiquadratus*
824	N	*Conus*	*aito*
825	N	*Conus*	*alfi*

826	N	*Conus*	*alrobini*
827	N	*Conus*	*amplus*
828	N	*Conus*	*annegretae*
829	N	*Conus*	*ariejoostei*
830	N	*Conus*	*aureopunctatus*
831	N	*Conus*	*brunneofilaris*
832	N	*Conus*	*caillaudi*
833	N	*Conus*	*cargilei*
834	N	*Conus*	*carioca*
835	N	*Conus*	*castaneus*
836	N	*Conus*	*ceruttii*
837	N	*Conus*	*cingulatus*
838	N	*Conus*	*colombianus*
839	N	*Conus*	*daphne*
840	N	*Conus*	*darkini*
841	N	*Conus*	*devorsinei*
842	N	*Conus*	*dispar*
843	N	*Conus*	*ednae*
844	N	*Conus*	*ernesti*
845	N	*Conus*	*evansi*
846	N	*Conus*	*flamingo*
847	N	*Conus*	*fonsecai*
848	N	*Conus*	*furnae*
849	N	*Conus*	*galeao*
850	N	*Conus*	*garciai*
851	N	*Conus*	*gibsonsmithorum*
852	N	*Conus*	*guanahacabibensis*
853	N	*Conus*	*hoaraui*
854	N	*Conus*	*honkerorum*
855	N	*Conus*	*indomaris*
856	N	*Conus*	*keatii*
857	N	*Conus*	*kerstitchi*

858	N	*Conus*	*kostini*
859	N	*Conus*	*largilliertii*
860	N	*Conus*	*malabaricus*
861	N	*Conus*	*malcolmi*
862	N	*Conus*	*mariaodeteae*
863	N	*Conus*	*minimus*
864	N	*Conus*	*moncuri*
865	N	*Conus*	*monilifer*
866	N	*Conus*	*moolenbeeki*
867	N	*Conus*	*mpenjatiensis*
868	N	*Conus*	*mulderi*
869	N	*Conus*	*nahoonensis*
870	N	*Conus*	*nocturnus*
871	N	*Conus*	*norai*
872	N	*Conus*	*nybakkeni*
873	N	*Conus*	*ostrinus*
874	N	*Conus*	*paraguana*
875	N	*Conus*	*paschalli*
876	N	*Conus*	*patglicksteinae*
877	N	*Conus*	*paulae*
878	N	*Conus*	*pauperculus*
879	N	*Conus*	*petestimpsoni*
880	N	*Conus*	*pica*
881	N	*Conus*	*pomareae*
882	N	*Conus*	*purissimus*
883	N	*Conus*	*quasimagus*
884	N	*Conus*	*regularis*
885	N	*Conus*	*richardsae*
886	N	*Conus*	*riosi*
887	N	*Conus*	*rosemaryae*
888	N	*Conus*	*ruthae*
889	N	*Conus*	*santaluziensis*

890	N	*Conus*	*scalaris*
891	N	*Conus*	*scalarissimus*
892	N	*Conus*	*sculletti*
893	N	*Conus*	*sennottorum*
894	N	*Conus*	*sinaiensis*
895	N	*Conus*	*skoglundae*
896	N	*Conus*	*smoesi*
897	N	*Conus*	*straturatus*
898	N	*Conus*	*sunderlandi*
899	N	*Conus*	*thevenardensis*
900	N	*Conus*	*tonisii*
901	N	*Conus*	*tristensis*
902	N	*Conus*	*urashimanus*
903	N	*Conus*	*vanvilstereni*
904	N	*Conus*	*velliesi*
905	N	*Conus*	*vezzaroi*
906	N	*Conus*	*vidua*
907	N	*Conus*	*xhosa*
908	N	*Profundiconus*	*barazeri*
909	N	*Profundiconus*	*cakobaui*
910	N	*Profundiconus*	*emersoni*
911	N	*Profundiconus*	*frausseni*
912	N	*Profundiconus*	*hennigi*
913	N	*Profundiconus*	*ikedai*
914	N	*Profundiconus*	*jeanmartini*
915	N	*Profundiconus*	*lani*
916	N	*Profundiconus*	*limpalaeri*
917	N	*Profundiconus*	*loyaltiensis*
918	N	*Profundiconus*	*maribelae*
919	N	*Profundiconus*	*neocaledonicus*
920	N	*Profundiconus*	*neotorquatus*
921	N	*Profundiconus*	*puillandrei*

922	N	*Profundiconus*	*robmoolenbeeki*
923	N	*Profundiconus*	*scopulicola*
924	N	*Profundiconus*	*smirnoides*
925	N	*Profundiconus*	*stahlschmidti*
926	N	*Profundiconus*	*tuberculosus*
927	N	*Profundiconus*	*virginiae*
928	N	*Profundiconus*	*zardoyai*
929	N	*Profundiconus*	*tarava*
930	N	*Profundiconus*	*weii*

Table S2 List of Conus species contained by the phylogenetic tree

No.	Genus	Species	No.	Genus	Species	No.	Genus	Species
1	*Californiconus*	*californicus*	113	*Conus*	*crotchii*	225	*Conus*	*mitratus*
2	*Profundiconus*	*kanakinus*	114	*Conus*	*cuneolus*	226	*Conus*	*moluccensis*
3	*Profundiconus*	*profundorum*	115	*Conus*	*curassaviensis*	227	*Conus*	*monachus*
4	*Profundiconus*	*smirna*	116	*Conus*	*curralensis*	228	*Conus*	*monile*
5	*Profundiconus*	*vaubani*	117	*Conus*	*cuvieri*	229	*Conus*	*moreleti*
6	*Conasprella*	*alisi*	118	*Conus*	*dalli*	230	*Conus*	*mozambicus*
7	*Conasprella*	*aphrodite*	119	*Conus*	*damottai*	231	*Conus*	*mucronatus*
8	*Conasprella*	*arcuata*	120	*Conus*	*daucus*	232	*Conus*	*muriculatus*
9	*Conasprella*	*articulata*	121	*Conus*	*dayriti*	233	*Conus*	*mus*
10	*Conasprella*	*baileyi*	122	*Conus*	*decoratus*	234	*Conus*	*musicus*
11	*Conasprella*	*boholensis*	123	*Conus*	*delanoyae*	235	*Conus*	*mustelinus*
12	*Conasprella*	*boucheti*	124	*Conus*	*denizi*	236	*Conus*	*namocanus*
13	*Conasprella*	*centurio*	125	*Conus*	*diadema*	237	*Conus*	*natalis*
14	*Conasprella*	*comatosa*	126	*Conus*	*diminutus*	238	*Conus*	*navarroi*
15	*Conasprella*	*coriolisi*	127	*Conus*	*distans*	239	*Conus*	*neptunus*
16	*Conasprella*	*delessertii*	128	*Conus*	*dorotheae*	240	*Conus*	*nigrescens*
17	*Conasprella*	*elokismenos*	129	*Conus*	*dorreensis*	241	*Conus*	*nigropunctatus*
18	*Conasprella*	*eucoronata*	130	*Conus*	*dusaveli*	242	*Conus*	*nimbosus*
19	*Conasprella*	*eugrammata*	131	*Conus*	*ebraeus*	243	*Conus*	*nobilis*
20	*Conasprella*	*guidopoppei*	132	*Conus*	*eburneus*	244	*Conus*	*nucleus*
21	*Conasprella*	*hopwoodi*	133	*Conus*	*echinophilus*	245	*Conus*	*nussatella*
22	*Conasprella*	*ichinoseana*	134	*Conus*	*emaciatus*	246	*Conus*	*nux*
23	*Conasprella*	*ione*	135	*Conus*	*episcopatus*	247	*Conus*	*obscurus*
24	*Conasprella*	*joliveti*	136	*Conus*	*ermineus*	248	*Conus*	*ochroleucus*
25	*Conasprella*	*kimioi*	137	*Conus*	*eversoni*	249	*Conus*	*omaria*
26	*Conasprella*	*longurionis*	138	*Conus*	*excelsus*	250	*Conus*	*orion*
27	*Conasprella*	*lucida*	139	*Conus*	*eximius*	251	*Conus*	*parius*
28	*Conasprella*	*mahogani*	140	*Conus*	*felitae*	252	*Conus*	*parvatus*
29	*Conasprella*	*mazei*	141	*Conus*	*fergusoni*	253	*Conus*	*parvulus*
30	*Conasprella*	*memiae*	142	*Conus*	*fernandesi*	254	*Conus*	*patricius*
31	*Conasprella*	*mindana*	143	*Conus*	*ferrugineus*	255	*Conus*	*pennaceus*
32	*Conasprella*	*orbignyi*	144	*Conus*	*figulinus*	256	*Conus*	*pergrandis*
33	*Conasprella*	*otohimeae*	145	*Conus*	*flavescens*	257	*Conus*	*pertusus*
34	*Conasprella*	*pagoda*	146	*Conus*	*flavidus*	258	*Conus*	*philippii*
35	*Conasprella*	*perplexa*	147	*Conus*	*flavus*	259	*Conus*	*pictus*
36	*Conasprella*	*pseudokimioi*	148	*Conus*	*floccatus*	260	*Conus*	*planorbis*
37	*Conasprella*	*pseudorbignyi*	149	*Conus*	*floridulus*	261	*Conus*	*plinthis*
38	*Conasprella*	*puncticulata*	150	*Conus*	*franciscanus*	262	*Conus*	*poormani*
39	*Conasprella*	*sieboldii*	151	*Conus*	*franciscoi*	263	*Conus*	*praecellens*
40	*Conasprella*	*stearnsii*	152	*Conus*	*freitasi*	264	*Conus*	*princeps*
41	*Conasprella*	*tornata*	153	*Conus*	*frigidus*	265	*Conus*	*proximus*
42	*Conasprella*	*viminea*	154	*Conus*	*fumigatus*	266	*Conus*	*pseudimperialis*
43	*Conasprella*	*wakayamaensis*	155	*Conus*	*furvus*	267	*Conus*	*pseudonivifer*
44	*Conasprella*	*ximenes*	156	*Conus*	*fuscoflavus*	268	*Conus*	*pulcher*
45	*Conus*	*abbreviatus*	157	*Conus*	*gauguini*	269	*Conus*	*pulicarius*

46	*Conus*	*achatinus*	158	*Conus*	*generalis*	270	*Conus*	*purpurascens*
47	*Conus*	*acutangulus*	159	*Conus*	*genuanus*	271	*Conus*	*queenslandis*
48	*Conus*	*alconnelli*	160	*Conus*	*geographus*	272	*Conus*	*quercinus*
49	*Conus*	*amadis*	161	*Conus*	*gladiator*	273	*Conus*	*radiatus*
50	*Conus*	*ammiralis*	162	*Conus*	*glans*	274	*Conus*	*rattus*
51	*Conus*	*amphiurgus*	163	*Conus*	*gloriamaris*	275	*Conus*	*raulsilvai*
52	*Conus*	*anabathrum*	164	*Conus*	*gondwanensis*	276	*Conus*	*recurvus*
53	*Conus*	*andamanensis*	165	*Conus*	*gonsaloi*	277	*Conus*	*regius*
54	*Conus*	*andremenezi*	166	*Conus*	*gradatus*	278	*Conus*	*regonae*
55	*Conus*	*anemone*	167	*Conus*	*grahami*	279	*Conus*	*retifer*
56	*Conus*	*angasi*	168	*Conus*	*grangeri*	280	*Conus*	*richardbinghami*
57	*Conus*	*antoniaensis*	169	*Conus*	*granum*	281	*Conus*	*richeri*
58	*Conus*	*antoniomonteiroi*	170	*Conus*	*guanche*	282	*Conus*	*roeckeli*
59	*Conus*	*araneosus*	171	*Conus*	*gubernator*	283	*Conus*	*rolani*
60	*Conus*	*arangoi*	172	*Conus*	*guinaicus*	284	*Conus*	*roseorapum*
61	*Conus*	*archon*	173	*Conus*	*hamamotoi*	285	*Conus*	*sandwichensis*
62	*Conus*	*ardisiaceus*	174	*Conus*	*hieroglyphus*	286	*Conus*	*sanguinolentus*
63	*Conus*	*arenatus*	175	*Conus*	*hirasei*	287	*Conus*	*senegalensis*
64	*Conus*	*aristophanes*	176	*Conus*	*hughmorrisoni*	288	*Conus*	*shikamai*
65	*Conus*	*ateralbus*	177	*Conus*	*immelmani*	289	*Conus*	*simonis*
66	*Conus*	*augur*	178	*Conus*	*imperialis*	290	*Conus*	*spectrum*
67	*Conus*	*aulicus*	179	*Conus*	*infinitus*	291	*Conus*	*sponsalis*
68	*Conus*	*aureus*	180	*Conus*	*infrenatus*	292	*Conus*	*spurius*
69	*Conus*	*auricomus*	181	*Conus*	*inscriptus*	293	*Conus*	*stercusmuscarum*
70	*Conus*	*aurisiacus*	182	*Conus*	*isabelarum*	294	*Conus*	*striatellus*
71	*Conus*	*australis*	183	*Conus*	*jacarusoi*	295	*Conus*	*striatus*
72	*Conus*	*balteatus*	184	*Conus*	*janus*	296	*Conus*	*striolatus*
73	*Conus*	*bandanus*	185	*Conus*	*josephinae*	297	*Conus*	*stupa*
74	*Conus*	*barthelemyi*	186	*Conus*	*jourdani*	298	*Conus*	*sugimotonis*
75	*Conus*	*bartschi*	187	*Conus*	*jucundus*	299	*Conus*	*sulcatus*
76	*Conus*	*belairensis*	188	*Conus*	*judaeus*	300	*Conus*	*sutanorcum*
77	*Conus*	*bengalensis*	189	*Conus*	*kinoshitai*	301	*Conus*	*suturatus*
78	*Conus*	*betulinus*	190	*Conus*	*kintoki*	302	*Conus*	*tabidus*
79	*Conus*	*biliosus*	191	*Conus*	*klemae*	303	*Conus*	*taeniatus*
80	*Conus*	*blanfordianus*	192	*Conus*	*koukae*	304	*Conus*	*tenuistriatus*
81	*Conus*	*boavistensis*	193	*Conus*	*laterculatus*	305	*Conus*	*terebra*
82	*Conus*	*boeticus*	194	*Conus*	*legatus*	306	*Conus*	*tessulatus*
83	*Conus*	*borgesi*	195	*Conus*	*lenavati*	307	*Conus*	*textile*
84	*Conus*	*bruguieresi*	196	*Conus*	*leopardus*	308	*Conus*	*thalassiarchus*
85	*Conus*	*brunneus*	197	*Conus*	*lischkeanus*	309	*Conus*	*thomae*
86	*Conus*	*bruuni*	198	*Conus*	*litoglyphus*	310	*Conus*	*tiaratus*
87	*Conus*	*bullatus*	199	*Conus*	*litteratus*	311	*Conus*	*tinianus*
88	*Conus*	*buxeus*	200	*Conus*	*lividus*	312	*Conus*	*tribblei*
89	*Conus*	*buxeus_loroisii*	201	*Conus*	*lobitensis*	313	*Conus*	*trochulus*
90	*Conus*	*byssinus*	202	*Conus*	*locumtenens*	314	*Conus*	*trovaoi*
91	*Conus*	*calhetae*	203	*Conus*	*lohri*	315	*Conus*	*tulipa*
92	*Conus*	*cancellatus*	204	*Conus*	*longilineus*	316	*Conus*	*unifasciatus*
93	*Conus*	*canonicus*	205	*Conus*	*lozeti*	317	*Conus*	*variegatus*

94	*Conus*	*capitanellus*	206	*Conus*	*luciae*	318	*Conus*	*varius*
95	*Conus*	*capitaneus*	207	*Conus*	*lugubris*	319	*Conus*	*ventricosus*
96	*Conus*	*caracteristicus*	208	*Conus*	*luteus*	320	*Conus*	*venulatus*
97	*Conus*	*catus*	209	*Conus*	*lynceus*	321	*Conus*	*verdensis*
98	*Conus*	*cedonulli*	210	*Conus*	*madecassinus*	322	*Conus*	*vexillum*
99	*Conus*	*cervus*	211	*Conus*	*magnificus*	323	*Conus*	*victoriae*
100	*Conus*	*chaldaeus*	212	*Conus*	*magus*	324	*Conus*	*villepinii*
101	*Conus*	*chiangi*	213	*Conus*	*maioensis*	325	*Conus*	*viola*
102	*Conus*	*chytreus*	214	*Conus*	*mappa*	326	*Conus*	*violaceus*
103	*Conus*	*cinereus*	215	*Conus*	*marmoreus*	327	*Conus*	*virgatus*
104	*Conus*	*circumactus*	216	*Conus*	*martensi*	328	*Conus*	*virgo*
105	*Conus*	*circumcisus*	217	*Conus*	*medoci*	329	*Conus*	*vittatus*
106	*Conus*	*cloveri*	218	*Conus*	*melvilli*	330	*Conus*	*vitulinus*
107	*Conus*	*coelinae*	219	*Conus*	*mercator*	331	*Conus*	*voluminalis*
108	*Conus*	*coffeae*	220	*Conus*	*micropunctatus*	332	*Conus*	*vulcanus*
109	*Conus*	*consors*	221	*Conus*	*miles*	333	*Conus*	*xicoi*
110	*Conus*	*corallinus*	222	*Conus*	*miliaris*	334	*Conus*	*zebroides*
111	*Conus*	*coronatus*	223	*Conus*	*miniexcelsus*	335	*Conus*	*zeylanicus*
112	*Conus*	*crocatus*	224	*Conus*	*miruchae*	336	*Conus*	*zonatus*
						337	*Conus*	*zylmanae*

Table S3 Raw data of the radar plot input table.

Species	Feeding Habits	Biosamples	Venom Glands	Genomes	Protein Structures	Clinical	Red List
Conus abbreviatus	Vermivore	X					
Conus acutangulus	Vermivore	X					
Conus adamsonii	Piscivore	X					
Conus aemulus	Vermivore	X					
Conus alconnelli	Vermivore	X					
Conus allaryi	-	X					X
Conus amadis	Molluscivore	X			X		
Conus ammiralis	Molluscivore	X					
Conus amphiurgus	Vermivore	X					
Conus anabathrum	Vermivore	X					X
Conus andremenezi	-	X					
Conus anemone	Vermivore	X					
Conus antoniomonteiroi	Vermivore	X	X				
Conus araneosus	Molluscivore	X			X		
Conus arangoi	Vermivore	X					
Conus archon	Vermivore	X					
Conus ardisiaceus	Vermivore						X
Conus arenatus	Vermivore	X	X				
Conus aristophanes	Vermivore	X					
Conus armadillo	Vermivore	X					
Conus ateralbus	Vermivore	X					X
Conus atlanticoselvagem	*						X
Conus augur	Vermivore	X					
Conus aulicus	Molluscivore	X			X		
Conus aurantius	-	X					X
Conus auricomus	Molluscivore	X					
Conus australis	Vermivore	X					
Conus balteatus	Vermivore	X					
Conus bandanus	Molluscivore	X					
Conus barthelemyi	Piscivore	X					

Conus bartschi	Vermivore	X					
Conus bayani	Vermivore	X	X				
Conus belairensis	Vermivore						X
Conus betulinus	Vermivore	X	X	X	X		
Conus biliosus	-	X	X				
Conus boavistensis	Vermivore	X	X				
Conus boeticus	Vermivore	X					
Conus borgesi	Vermivore	X					
Conus boschorum	*						X
Conus boui	Vermivore	X					
Conus bruguieresi	Vermivore						X
Conus brunneus	Vermivore	X					
Conus bruuni	Vermivore / Piscivore	X					
Conus bullatus	Piscivore / Molluscivore	X		X	X		
Conus burryae	Vermivore	X					
Conus buxeus	Vermivore				X		
Conus byssinus	Vermivore	X					
Conus cacao	*						X
Conus cancellatus	Vermivore	X					
Conus canonicus	Molluscivore	X					
Conus capitanellus	Vermivore	X					
Conus capitaneus	Vermivore	X					
Conus caracteristicus	Vermivore	X					
Conus cardinalis	-	X					X
Conus catus	Piscivore	X				X	
Conus cedonulli	Vermivore	X					
Conus cepasi	-						X
Conus chaldaeus	Vermivore	X					
Conus chiangi	Vermivore	X					
Conus cinereus	Piscivore	X					
Conus circumactus	-	X					
Conus circumcisus	Piscivore	X					
Conus cloveri	Vermivore						X

Conus cocceus	Vermivore	X					
Conus coelinae	Vermivore	X					
Conus coffeae	Vermivore	X					
Conus compressus	Vermivore						X
Conus consors	Piscivore	X	X	X	X		
Conus corallinus	Vermivore	X					
Conus coronatus	Vermivore	X	X				
Conus crotchii	Vermivore	X					X
Conus cuneolus	Vermivore	X	X				X
Conus curassaviensis	Vermivore	X					X
Conus curralensis	Vermivore						X
Conus cuvieri	Piscivore	X					X
Conus dalli	Molluscivore	X					
Conus damottai	Vermivore	X					
Conus darkini	-	X					
Conus daucus	Vermivore	X					
Conus dayriti	Vermivore	X					
Conus decoratus	Vermivore	X					X
Conus delanoyae	Vermivore	X					
Conus denizi	Vermivore						X
Conus derrubado	*						X
Conus diadema	Vermivore	X					
Conus diminutus	Vermivore	X					X
Conus distans	Vermivore	X					
Conus dorotheae	Vermivore	X					X
Conus dorreensis	Vermivore	X					
Conus duffyi	-						X
Conus ebraeus	Vermivore	X	X				
Conus eburneus	Vermivore / Piscivore	X					
Conus echinophilus	Vermivore						X
Conus emaciatus	Vermivore	X					
Conus encaustus	-	X					
Conus episcopatus	Molluscivore	X	X		X		

Conus ermineus	Piscivore	X	X		X		
Conus erythraeensis	Vermivore / Piscivore	X					
Conus evorai	*						X
Conus excelsus	Vermivore	X					
Conus explorator	-						X
Conus felitae	Vermivore						X
Conus fergusoni	Vermivore	X					
Conus fernandesi	Vermivore						X
Conus figulinus	Vermivore	X					
Conus fijisulcatus	Vermivore	X					
Conus flavidus	Vermivore	X					
Conus floccatus	Piscivore	X					
Conus floridulus	Vermivore	X					
Conus fontonae	*						X
Conus frigidus	Vermivore	X					
Conus fumigatus	-	X					
Conus furvus	Molluscivore	X					
Conus fuscoflavus	Vermivore	X					
Conus galeao	-	X	X				
Conus gauguini	Piscivore	X					X
Conus generalis	Vermivore	X			X		
Conus geographus	Piscivore	X	X		X	X	
Conus gigasulcatus	Vermivore	X					
Conus gladiator	Vermivore	X					
Conus glans	Vermivore	X					
Conus glaucus	Vermivore	X					
Conus gloriamaris	Molluscivore	X	X		X		
Conus gondwanensis	Vermivore	X					
Conus grahami	Vermivore	X	X				
Conus granulatus	Vermivore / Piscivore	X					
Conus granum	Vermivore	X					
Conus guanche	Vermivore	X	X				
Conus gubernator	Piscivore	X					

Conus guinaicus	Vermivore						X
Conus hamamotoi	-	X					
Conus henckesi	-						X
Conus hennequini	Vermivore						X
Conus hieroglyphus	Vermivore	X					X
Conus hilli	*	X					
Conus hirasei	Vermivore	X					
Conus hughmorrisoni	-	X					
Conus hybridus	*	X					X
Conus immelmani	Molluscivore						X
Conus imperialis	Vermivore	X	X		X		
Conus infinitus	Vermivore	X	X				
Conus inscriptus	Vermivore				X		
Conus jacarusoi	-	X					
Conus janus	Vermivore	X					
Conus jeanmartini	-						X
Conus josephinae	Vermivore						X
Conus judaeus	Vermivore	X					
Conus julii	Piscivore						X
Conus kermadecensis	Vermivore	X					
Conus kersteni	Vermivore						X
Conus kinoshitai	Vermivore / Piscivore				X		
Conus kintoki	Vermivore	X					
Conus kirkandersi	-						X
Conus klemae	Vermivore	X					
Conus kuroharai	Vermivore	X					
Conus laterculatus	Vermivore / Piscivore	X					
Conus legatus	Molluscivore	X					
Conus lenavati	Vermivore	X	X				
Conus leopardus	Vermivore	X					
Conus lineopunctatus	-	X					
Conus lischkeanus	Vermivore	X					
Conus litoglyphus	Vermivore	X					

Conus litteratus	Vermivore	X	X				
Conus lividus	Vermivore	X	X		X		
Conus locumtenens	Molluscivore	X					
Conus longilineus	Vermivore	X					
Conus lozeti	Vermivore	X					
Conus luciae	-	X					
Conus lugubris	Vermivore						X
Conus luquei	*						X
Conus madagascariensis	Molluscivore	X					
Conus madecassinus	Vermivore	X					
Conus magellanicus	-	X					
Conus magnificus	Molluscivore	X					
Conus magus	Piscivore	X	X		X	X	
Conus maioensis	Vermivore	X	X				
Conus maldivus	-	X					
Conus mappa	Vermivore / Molluscivore	X					
Conus marchionatus	Molluscivore	X					
Conus marmoreus	Molluscivore	X	X		X	X	
Conus martensi	Vermivore	X					
Conus massemini	*	X					
Conus mcbridei	Vermivore	X					
Conus medoci	Vermivore	X					
Conus melvilli	Vermivore						X
Conus mercator	Vermivore						X
Conus micropunctatus	-	X					
Conus miles	-	X			X		
Conus miliaris	Vermivore	X	X				
Conus miniexcelsus	-	X					
Conus miruchae	Vermivore	X	X				
Conus moluccensis	Vermivore / Piscivore	X					
Conus monachus	Piscivore	X					
Conus moncuri	-	X					
Conus monile	Vermivore	X			X		

Conus mordeirae	*			X
Conus moreleti	Vermivore	X		
Conus mucronatus	Piscivore	X		
Conus muriculatus	Vermivore	X		
Conus mus	Vermivore	X		
Conus musicus	Vermivore	X		
Conus mustelinus	Vermivore	X		
Conus namocanus	Vermivore	X		
Conus navarroi	Vermivore			X
Conus nielsenae	Vermivore			X
Conus nimbosus	-	X		
Conus nobilis	Vermivore / Molluscivore	X		
Conus nobrei	-			X
Conus nussatella	Vermivore / Molluscivore	X		
Conus nux	Vermivore	X	X	
Conus obscurus	Piscivore	X		
Conus ochroleucus	Vermivore	X		
Conus omaria	Molluscivore	X	X	
Conus orion	Vermivore	X		
Conus papilliferus	Vermivore	X		
Conus parius	Piscivore	X		
Conus patricius	Vermivore	X		
Conus pennaceus	Molluscivore		X	
Conus pergrandis	Vermivore / Piscivore	X		
Conus pertusus	Vermivore	X		
Conus pictus	Vermivore		X	
Conus pineaui	*	X		
Conus planorbis	Vermivore	X	X	
Conus plinthis	Vermivore	X		
Conus poormani	Vermivore	X		
Conus praecellens	Vermivore	X		
Conus princeps	Vermivore	X		
Conus proximus	Piscivore	X		

Conus pseudimperialis	-	X					
Conus purpurascens	Piscivore	X			X		
Conus queenslandis	Vermivore	X					
Conus quercinus	Vermivore	X	X				
Conus radiatus	Vermivore / Piscivore				X		
Conus rattus	Vermivore	X	X				
Conus raulsilvai	Vermivore	X	X				
Conus rawaiensis	-						X
Conus recurvus	Vermivore	X					
Conus regius	Vermivore	X			X		
Conus regonae	Vermivore	X					X
Conus retifer	Molluscivore	X					
Conus richardbinghami	-	X					X
Conus rolani	Vermivore / Piscivore	X			X		
Conus sakalava	*	X					
Conus salreiensis	*						X
Conus sanguinolentus	Vermivore	X					
Conus saragasae	Vermivore						X
Conus shikamai	Vermivore	X					
Conus sponsalis	Vermivore	X	X				
Conus spurius	Vermivore	X					
Conus spurius	Vermivore	X					
Conus stearnsii	Vermivore						X
Conus stercusmuscarum	Piscivore	X			X		
Conus stimpsoni	Vermivore	X					
Conus striatellus	Vermivore	X					
Conus striatus	Piscivore	X	X		X		
Conus striolatus	Piscivore	X			X		
Conus stupa	-	X					
Conus sugimotonis	Vermivore	X					
Conus sulcatus	Vermivore	X					
Conus sunderlandi	-	X					
Conus sutanorcum	Piscivore	X					

Conus tabidus	Vermivore	X					
Conus tacomae	Vermivore						X
Conus taeniatus	Vermivore	X					
Conus taslei	*						X
Conus teodorae	*						X
Conus terebra	Vermivore	X	X				
Conus terryni	Vermivore						X
Conus tessulatus	Vermivore / Piscivore	X					
Conus textile	Molluscivore	X	X		X		
Conus thevenardensis	*						X
Conus tinianus	Vermivore	X					
Conus trencarti	Vermivore						X
Conus tribblei	Vermivore	X	X	X			
Conus trigonus	Vermivore	X					
Conus trochulus	Vermivore	X	X				X
Conus troendlei	-	X					
Conus tulipa	Piscivore	X			X	X	
Conus unifasciatus	Vermivore						X
Conus variegatus	-	X					
Conus varius	Vermivore	X	X				
Conus vautieri	-	X					
Conus ventricosus	Vermivore	X		X			
Conus venulatus	Vermivore	X					
Conus verdensis	Vermivore	X	X				
Conus vexillum	Vermivore	X	X				
Conus vezoi	-	X					
Conus victoriae	Molluscivore	X	X		X	X	
Conus villepinii	Vermivore	X			X		
Conus viola	Vermivore	X					
Conus virgatus	Vermivore	X					
Conus virgo	Vermivore	X	X				
Conus vittatus	Vermivore	X					
Conus voluminalis	Vermivore	X					

Conus xicoi	Vermivore	X					X
Conus zebroides	-						X
Conus zeylanicus	Vermivore	X					
Conus zonatus	Vermivore	X			X		
Conus zylmanae	-	X					

Table S4 Input data for the radar plot

	Vermivorous (V)	Molluscivous (M)	Piscivorous (P)	lack of data	Sum
**Genome Assembly**	3	1	1	0	5
**Species went clinical trial**	0	2	4	0	6
**Protein Structure**	16	11	13	1	37
**Venom Gland**	31	5	5	2	43
**Species numbers (overlaps are counted separately)**	368	61	100	412	506
**Red list**	37	1	3	26	67
